# Normative Data on Serum and Plasma Tryptophan and Kynurenine Concentrations from 8089 Individuals Across 120 Studies: A Systematic Review and Meta-Analysis

**DOI:** 10.1177/11786469231211184

**Published:** 2023-11-29

**Authors:** Najwa-Joelle Metri, Ali S Butt, Ava Murali, Genevieve Z Steiner-Lim, Chai K Lim

**Affiliations:** 1 NICM Health Research Institute, Western Sydney University, Penrith, NSW, Australia; 2 Translational Health Research Institute (THRI), Western Sydney University, Penrith, NSW, Australia; 3 Faculty of Medicine, Health and Human Sciences, Macquarie University, Macquarie Park, NSW, Australia

**Keywords:** Tryptophan, kynurenine, normative data, metabolomics, meta-analysis, systematic review

## Abstract

In this systematic review and meta-analysis, a normative dataset is generated from the published literature on the kynurenine pathway in control participants extracted from case-control and methodological validation studies. Study characteristics were mapped, and studies were evaluated in terms of analytical rigour and methodological validation. Meta-analyses of variance between types of instruments, sample matrices and metabolites were conducted. Regression analyses were applied to determine the relationship between metabolite, sample matrix, biological sex, participant age and study age. The grand mean concentrations of tryptophan in the serum and plasma were 60.52 ± 15.38 μM and 51.45 ± 10.47 μM, respectively. The grand mean concentrations of kynurenine in the serum and plasma were 1.96 ± 0.51 μM and 1.82 ± 0.54 μM, respectively. Regional differences in metabolite concentrations were observed across America, Asia, Australia, Europe and the Middle East. Of the total variance within the data, mode of detection (MOD) accounted for up to 2.96%, sample matrix up to 3.23%, and their interaction explained up to 1.53%; the latter of which was determined to be negligible. This review was intended to inform future empirical research and method development studies and successfully synthesised pilot data. The pilot data reported in this study will inform future precision medicine initiatives aimed at targeting the kynurenine pathway by improving the availability and quality of normative data.

## Introduction

Tryptophan (TRP) metabolism and activity in the kynurenine pathway (KP) have been implicated in an array of conditions, including depressive, psychotic,^1^ and metabolic disorders,^2^ neurodegenerative,^3^ autoimmune,^4^ cardiovascular,^5^ kidney,^6^ and gastrointestinal diseases^7^ and cancer.^8^ This is due to their widespread roles in modulating both innate and adaptive immune responses and inflammation.^9,10^ As a result, it has been suggested that the thorough analytical profiling of KP metabolites may improve patient care outcomes by revealing prognostic, diagnostic and theragnostic biomarkers.^11^

Several meta-analyses have extensively profiled KP metabolites across various clinical cohorts within the plasma, serum, urine and cerebrospinal fluid.^1,12-15^ Examples of the variables summarised include modes of detection (MODs) and relevant instrumentation, metabolites studied, sample matrices and geographical locations. Despite the breadth of these data, there is a lack of information on healthy and younger cohorts (eg, aged ⩽ 50 years) with normal metabolic functioning. Inter-sample variation in KP profiling is also less characterised. For example, studies have found that the information contained in peripheral samples, such as serum and urine, are statistically independent.^16^ Changes in the KP profile across the lifespan are even less characterised. These literature gaps are emphasised by the heterogeneity in methodological and analytical quality of the reported literature. Together, these factors mean that there is an absence of normative KP data, across the lifespan and across geographical regions, that is of high methodological and analytical rigour that may be referred to as a benchmark in future studies, clinical or otherwise.

The purpose of this study was to generate a set of KP normative values from the existing literature for the purpose of informing future biomarker investigations. This meta-analysis and systematic review therefore aimed to: (i) map the characteristics of studies reported within the literature, including MOD, metabolites studied, sample matrices and geographical location; (ii) calculate normative means for TRP and kynurenine (KYN) across these characteristics; (iii) critically appraise the methodologies and data reported within the literature, in terms of author-reported methodological validation and through an independent risk of bias assessment; (iv) ascertain variance in sensitivity between MOD; and (v) determine the relationship between metabolite, biofluid, age, sex and study year.

## Methods and Materials

### Study protocol

This systematic review and meta-analysis was prospectively registered with the PROSPERO International Database of Systematic Reviews on 26 December 2021 (#CRD42021293595), and followed the recommendations of the Preferred Reporting Items for Systematic Reviews and Meta-Analyses Statement (PRISMA).^17^ A review protocol was not prepared. It was anticipated that the review would provide insight into the evidence required to inform the outcomes of biomarker development research.

### Search strategy

Initially, a scoping search was conducted using a comprehensive strategy to establish the breadth of the review on 15 November 2021. Based on this, a comprehensive search of articles was conducted on 4 electronic databases comprising PubMed, Web of Science, EMBASE and Scopus. Databases were searched for articles from peer-reviewed journals from the databases’ date of inception through to 27 December 2021. An example of the search algorithm from PubMed is described in Table 1. The same Medical Subject Headings (MeSH) and search strategy were applied across all 4 databases. However, minor adjustments were applied to allow for database-specific variations in the requirements for the search queries. Database alerts were set up to notify the authors of any new publications up until 1 October 2022 to allow for review finalisation, and citation searches on any reviews identified were performed. Authors were not contacted for missing data. Studies that did not report TRP or KYN as numerical values were excluded during the full-text screening phase.

**Table 1.** Population, Intervention, Comparison, Outcomes, Study Design (PICOS) framework illustrating the systematic search strategy for PubMed.

Interest
The use of liquid chromatography, mass spectrometry, or ELISA assay techniques. MeSH terms or other synonyms included ‘Chromatography, Liquid’ OR ‘Mass Spectrometry’ OR ‘Enzyme-Linked Immunosorbent Assay’ OR ‘ELISA’
Outcome
The identification and quantification of tryptophan or kynurenine in human biofluids. MeSH terms or other synonyms included ‘Tryptophan’ OR ‘Kynurenine’
Study design
Observational, cross-sectional case-control study. Data relating to the biofluids of healthy control groups will be extracted from clinical studies, or from analytical studies focussed on healthy groups only. MeSH terms or other synonyms included ‘Blood’ OR ‘Serum’ OR ‘Plasma’ OR ‘Urine’ OR ‘Saliva’ OR ‘Sebum’ OR ‘Tears’ OR ‘Sweat’ OR ‘Cerebrospinal Fluid’ OR ‘Feces’ OR ‘Faeces’

Interest, outcome and study design searches were combined with the Boolean operator ‘AND’. Comparison terms were not specified due to the broad nature of the review.

### Study selection

In total, 11 241 records were obtained from the systematic database search, indicating the feasibility of the review. All included studies were exported to a reference management software (EndNote™ X9; Thomson Reuters, CA, USA). Using EndNote, duplicate references were removed, and remaining titles and abstracts were screened against study inclusion and exclusion criteria by the primary reviewer (NJM). The titles and abstracts of the remaining records were screened and if there was any doubt regarding their eligibility, the full text was retrieved for clarification. Twenty percent of articles deemed eligible to the primary reviewer were then confirmed by 2 independent reviewers (CKL & GZS) to ensure inclusion criteria were met.

### Eligibility criteria

Inclusion criteria for clinical and/or analytical studies included: (i) quantifying KP metabolites TRP and/or KYN in human biofluids including whole blood, blood serum, blood plasma, urine, saliva, sebum, tears, sweat, cerebrospinal fluid and faeces via targeted metabolomic approaches; (ii) observational cross-sectional case-control studies which included a healthy control group or analytical studies including only healthy adults with no known neurological, psychological or medical condition; (iii) reported in the English language; and (iv) using liquid chromatography (LC) coupled to mass spectrometry (MS), electron capture dissociation (ECD), spectroscopy, or enzyme-linked immunosorbent assays (ELISA).

Exclusion criteria for publications included: (i) conference proceedings and papers, editorials, letters, notes, short surveys, pre-prints, book chapters and book series; (ii) publications that explore TRP or KYN identification and/or quantification in nutritional supplements, animals, food products, or following an intervention such as TRP loading or depletion; and (iii) studies which utilise untargeted metabolomics due to potential imprecision in the metabolites characterised.

### Data extraction

Data extraction was then completed (NJM, ASB, AM) and double-checked by a second independent reviewer (CKL, GZS, NJM, ASB, AM). Any disagreements were resolved by reviewing and discussing the articles amongst all co-authors (CKL, GZS, NJM, ASB, AM). Study characteristics were extracted including author, year of publication, MOD as per the instrument of detection, and the number of samples analysed. Concentrations of TRP and/or KYN in the biofluids of healthy adults were also extracted. In case-control studies, only data related to the control group were extracted and reported. The chromatographic conditions were extracted from eligible studies into standardised tables. To ensure the analytical rigour of the data, validation parameters of interest included: (i) determining the robustness of the method by testing the variation in the instrumental parameters; (ii) limits of quantification (LOQ); (iii) limits of detection (LOD); (iv) precision; (v) accuracy of the method via recoveries for determining true values; (vi) assessment of the analyte’s stability; (vii) calibration curve to test the response for a variety of standards; and (viii) the specificity such as matching the retention times of the samples and the standards.^18^

### Risk of bias evaluation

As not one appropriate tool was available to assess the methodological quality of studies, this was evaluated using a combination of tools, including the QUADOMICS tool,^19^ the QUADAS tool^20^ and the Joanna Briggs Institute (JBI) Checklist for Analytical Cross Sectional Studies.^21^ The adapted tool consisted of 6 items which account for the particular methodological challenges posed by metabolomic approaches (Supplemental Table S1). Items were either scores as Yes, No or NA/Unknown. Studies were then given an overall appraisal as either high quality or low quality by 3 members of the authorship team (NJM, ASB, AM).

### Statistical analyses

Studies included in the meta-analysis (serum and plasma) were grouped via the MOD as per instrument of detection, by metabolite (TRP or KYN), by sample matrix and by geographical region (America, Asia, Australia, Europe, Middle East). Findings from pooled data were compared by utilising established estimation and approximation methods reported in the literature,^22^ this was largely related to the conversion of median and interquartile range to mean and standard deviation (SD). Weighted mean (±SD) concentrations of TRP and KYN were calculated per MOD, sample matrix and by geographical region reported. Variance in sensitivity between instruments was ascertained by a 2-way ANOVA followed by Tukey’s multiple comparison test. Only sample matrices with over 20 studies reported were included into the meta-analysis to ensure that there was enough statistical power to detect an effect. Sample matrices with <20 studies reported were synthesised and reported descriptively, with weighted means and pooled SDs from non-blood biofluids computed and summarised accordingly. To determine the relationship between metabolite, biofluid, age, sex and study year, an adjusted weighted-variance Ordinary Least Squares (OLS) regression analysis was performed. All statistical analyses were performed using GraphPad Prism v 9.0.0 (GraphPad Software, Inc., CA, USA) and Stata™ v17 (StataCorp, Texas, USA).

## Results

**Figure 1.**
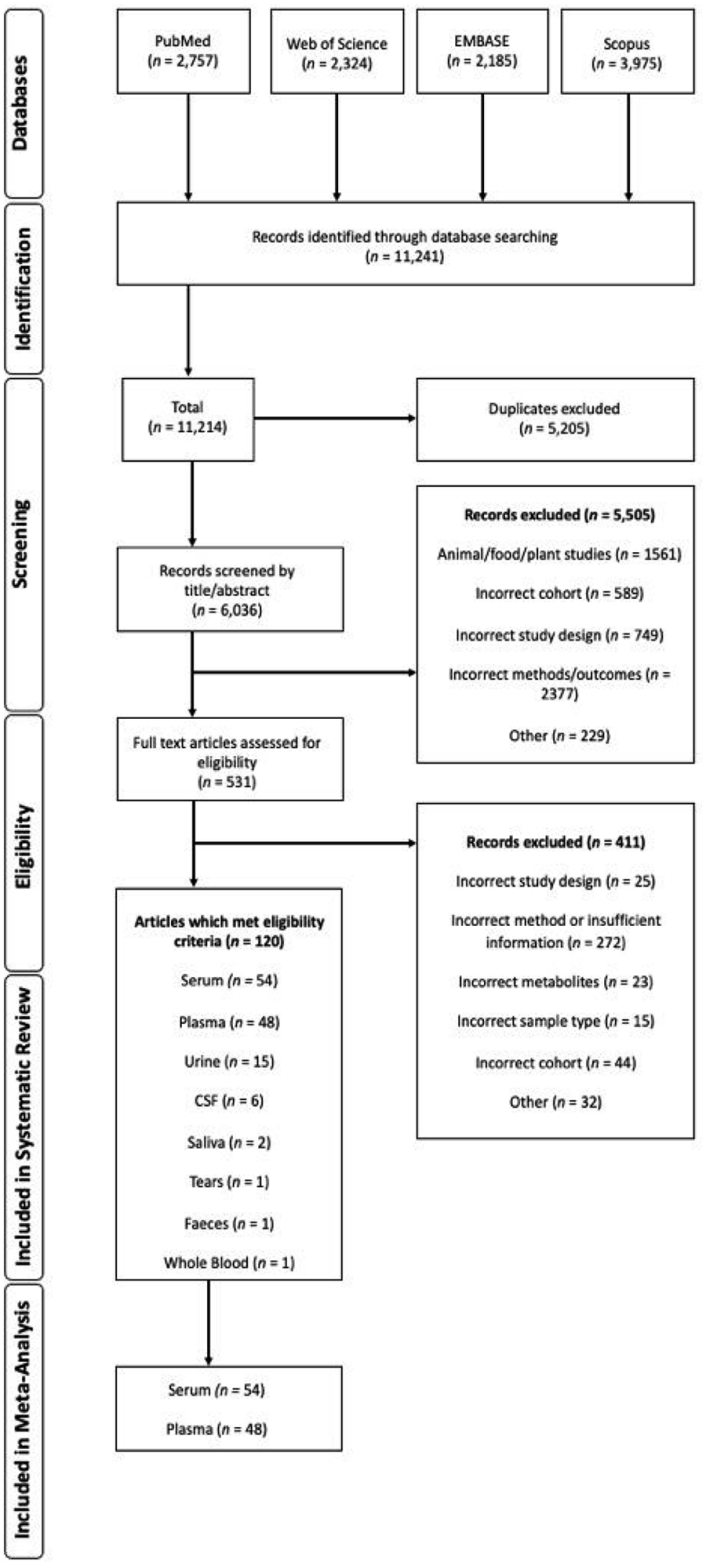
Preferred Reporting Items for Systematic Reviews and Meta-Analyses (PRISMA) flow diagram detailing the search, identification, screening and eligibility processes.

The search strategy identified 11 241 publications. Following the application of the criteria, 531 full-text articles were assessed, of which 22.60% (N = 120) met the inclusion criteria (Figure 1). Serum and plasma were the only sample matrices with over 20 studies reported for subsequent inclusion in the meta-analysis. Due to small sample sizes (and consequently a high level of variability), urine, CSF, saliva, tears, faeces and whole blood studies were not synthesised descriptively and not meta-analysed.

### Study characteristics

This meta-analysis reported on a grand total of 120 studies including 8089 participants (N = 120; n = 8089; Supplemental Table S2). Studies were reported from across 5 regions, including America, Asia, Australia, Europe and Middle East and 31 countries in total. Studies were most reported from China (23.33%; n = 28). The mean age across all studies was 47.35 ± 15.65 and of participants were female.

Regarding the quantification of TRP, a total of 8 sample matrices were reported within the literature. This included serum (n = 54), plasma (n = 48), urine (n = 15), cerebrospinal fluid (CSF; n = 6), saliva (n = 2), tears (n = 1), faeces (n = 1) and whole blood (n = 1). A total of 6 sample types were reported in the quantification of KYN: serum (n = 34), plasma (n = 33), urine (n = 7), CSF (n = 6), saliva (n = 2) and faeces (n = 1). The most common MOD across metabolites reported within the literature included mass spectrometry (MS; n = 80). Other modes of detection reported include fluorescence detection (FL), ultra-violet visible spectroscopy (UV-Vis), ELISA, photo diode array (PDA), ECD and electrochemiluminescence (ECL). The characteristics of the TRP and KYN quantification methods used in the included studies are described in Supplemental Table S3, including MOD, column phase and diameter, particle size, mobile phases, flow rate, injection volume, column temperature, programme time and detection wavelength (where applicable).

### Risk of bias evaluation and method validation

Risk of bias varied across the reported studies and the breakdown of the appraisal is detailed for each study and item in Table 2. All citations are listed in Table 2. Over half of the included studies clearly defined their criteria for inclusion (59.17%; n = 71), while only 28.33% of studies (n = 34) described the study subjects and setting in detail. Majority of studies sufficiently described the type of sample (65.83%; n = 79) and the handling and pre-analytical processing of the samples (72.5%; n = 87). Only 34.17% (n = 41) of included studies sufficiently described their methodological conditions. Indeed, 84.17% (*n* = 101) of included studies reported their statistical analysis. Overall, 51.67% (n = 62) of studies were subjectively appraised to be of high quality.

**Table 2.** Risk of bias across evaluation of included studies across the 6 bias domains and their overall rating.

Citation	Item 1	Item 2	Item 3	Item 4	Item 5	Item 6	Overall
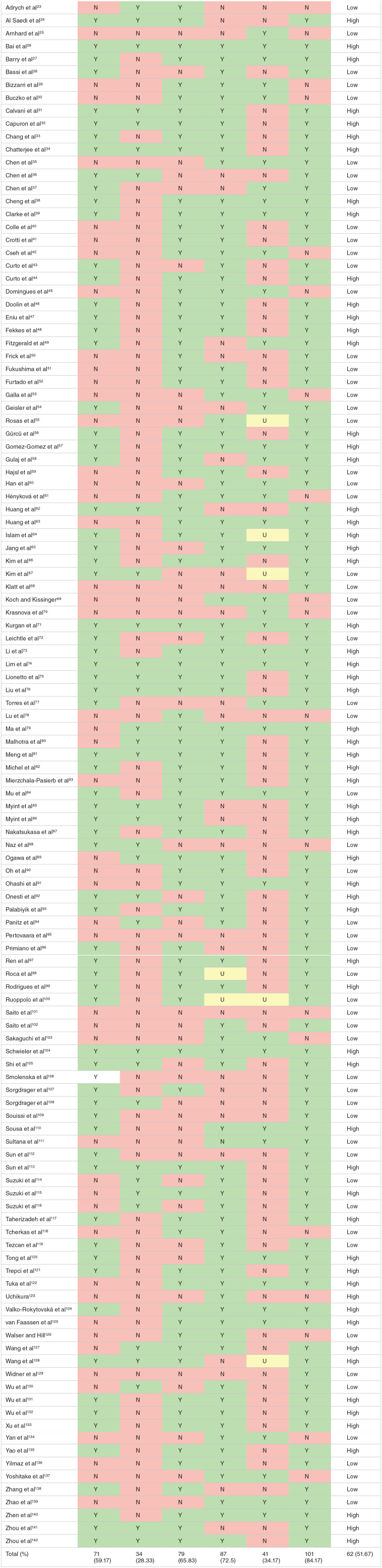

Abbreviations: N, no; U, unclear; Y, yes. Colours: N, red; U, yellow; Y, green.

Item 1: Were the criteria for inclusion in the sample clearly defined?

Item 2: Were the study subjects and the setting described in detail?

Item 3: Was the sample type used fully described?

Item 4: Were the handling of specimens and pre-analytical procedures reported in sufficient detail and similar for the whole sample?

Item 5: Was the execution of the index test described in sufficient detail to permit replication of the test?

Item 6: Were uninterpretable or intermediate test results reported?

Reporting of independent method validation was highly varied (Table 3). Overall, 80.83% (n = 73) of studies reported at least one parameter of their independent methodological validation. Most commonly, LOD was reported in 44.17% (n = 53) studies. This was closely followed by the presentation of calibration curves (39.17%; n = 47). Robustness of the methodology was least commonly validated in only 10.83% (n = 13) of studies.

**Table 3.** Reported method validation parameters of included studies, including specificity, calibration curve (CC), stability, accuracy, precision, LOD, LOQ and robustness.

Citation	Specificity	CC	Stability	Accuracy	Precision	LOD	LOQ	Robustness	Total (%)
Adrych et al, 2010	NR	NR	NR	NR	NR	NR	NR	NR	0 (0.00)
Al Saedi et al, 2022	NR	NR	NR	NR	NR	NR	NR	NR	0 (0.00)
Arnhard et al, 2018	**✓**	**✓**	**✓**	**✓**	**✓**	**✓**	**✓**	NR	7 (87.50)
Bai et al, 2021	**✓**	NR	NR	NR	NR	NR	NR	NR	1 (12.50)
Barry et al, 2009	NR	NR	NR	NR	NR	NR	NR	NR	0 (0.00)
Bassi et al, 2017	NR	NR	NR	NR	NR	NR	NR	NR	0 (0.00)
Bizzarri et al, 1990	NR	**✓**	NR	**✓**	NR	**✓**	NR	NR	3 (37.50)
Buczko et al, 2007	NR	NR	NR	NR	NR	NR	NR	NR	0 (0.00)
Calvani et al, 2020	NR	NR	NR	NR	NR	NR	NR	NR	0 (0.00)
Capuron et al, 2011	NR	NR	NR	**✓**	NR	NR	NR	NR	1 (12.50)
Chang et al, 2018	NR	NR	NR	NR	NR	NR	NR	NR	0 (0.00)
Chatterjee et al, 2019	**✓**	**✓**	NR	NR	NR	NR	NR	NR	2 (25.00)
Chen et al, 2019	**✓**	**✓**	**✓**	**✓**	**✓**	NR	**✓**	NR	6 (75.00)
Chen et al, 2010	NR	NR	NR	NR	NR	NR	NR	NR	0 (0.00)
Chen et al, 2020	NR	**✓**	**✓**	**✓**	**✓**	NR	**✓**	**✓**	6 (75.00)
Cheng et al, 2015	NR	**✓**	**✓**	**✓**	**✓**	**✓**	**✓**	NR	6 (75.00)
Clarke et al, 2009	NR	NR	NR	NR	NR	NR	NR	NR	0 (0.00)
Colle et al, 2020	NR	NR	NR	NR	NR	NR	NR	NR	0 (0.00)
Crotti et al, 2019	NR	NR	NR	NR	NR	NR	NR	NR	0 (0.00)
Cseh et al, 2019	NR	**✓**	NR	**✓**	**✓**	**✓**	**✓**	NR	5 (62.50)
Curto et al, 2016	NR	NR	NR	**✓**	**✓**	**✓**	**✓**	NR	4 (50.00)
Curto et al, 2016	NR	NR	NR	**✓**	**✓**	**✓**	**✓**	NR	4 (50.00)
Domingues et al, 2015	NR	**✓**	NR	**✓**	**✓**	NR	**✓**	NR	4 (50.00)
Doolin et al, 2018	NR	NR	NR	NR	NR	NR	**✓**	NR	1 (12.50)
Eniu et al, 2019	**✓**	**✓**	NR	NR	NR	**✓**	**✓**	NR	2 (25.00)
Fekkes et al, 1998	NR	NR	NR	NR	NR	NR	NR	NR	0 (0.00)
Fitzgerald et al, 2008	NR	NR	NR	NR	NR	NR	NR	NR	0 (0.00)
Frick et al, 2004	NR	NR	NR	NR	NR	NR	NR	NR	0 (0.00)
Fukushima et al, 2014	**✓**	NR	NR	NR	NR	NR	NR	NR	1 (12.50)
Furtado et al, 2017	NR	**✓**	NR	**✓**	**✓**	**✓**	**✓**	NR	5 (62.50)
Galla et al, 2021	**✓**	**✓**	**✓**	**✓**	**✓**	**✓**	**✓**	NR	7 (87.50)
Geisler et al, 2015	NR	NR	NR	NR	NR	NR	NR	NR	0 (0.00)
Gevorkian et al, 2015	**✓**	NR	NR	NR	NR	NR	NR	NR	1 (12.50)
Girgin et al, 2020	NR	NR	NR	NR	NR	NR	NR	NR	0 (0.00)
Gomez-Gomez et al, 2017	NR	NR	NR	NR	NR	NR	NR	NR	0 (0.00)
Gulaj et al, 2010	NR	NR	NR	NR	NR	**✓**	NR	NR	1 (12.50)
Hajsl et al, 2020	**✓**	**✓**	NR	**✓**	**✓**	NR	NR	NR	4 (50.00)
Han et al, 2018	**✓**	**✓**	**✓**	**✓**	**✓**	**✓**	NR	NR	6 (75.00)
Henykova et al, 2016	**✓**	NR	**✓**	**✓**	**✓**	**✓**	**✓**	NR	6 (75.00)
Huang et al, 2022	NR	NR	NR	NR	NR	NR	NR	NR	0 (0.00)
Huang et al, 2021	NR	**✓**	NR	**✓**	**✓**	**✓**	**✓**	NR	5 (62.50)
Islam et al, 2020	NR	NR	NR	NR	NR	NR	NR	NR	0 (0.00)
Jang et al, 2022	NR	**✓**	NR	NR	NR	**✓**	**✓**	NR	3 (37.50)
Kim et al, 2015	NR	NR	NR	NR	NR	NR	NR	NR	0 (0.00)
Kim et al, 2009	NR	**✓**	NR	NR	NR	NR	NR	NR	1 (12.50)
Klatt et al, 2021	NR	NR	NR	**✓**	NR	NR	NR	NR	1 (12.50)
Koch et al, 1979	NR	**✓**	NR	NR	**✓**	NR	NR	NR	2 (25.00)
Krasnova et al, 2000	NR	NR	**✓**	NR	NR	**✓**	NR	NR	2 (25.00)
Kurgan et al, 2022	NR	NR	NR	NR	NR	**✓**	**✓**	NR	2 (25.00)
Leichtle et al, 2012	NR	NR	NR	NR	NR	NR	NR	NR	0 (0.00)
Li et al, 2011	**✓**	**✓**	NR	NR	**✓**	**✓**	NR	NR	4 (50.00)
Lim et al, 2017	NR	NR	NR	NR	NR	NR	NR	NR	0 (0.00)
Lionetto et al, 2021	**✓**	NR	NR	NR	NR	NR	NR	NR	1 (12.50)
Liu et al, 2018	**✓**	**✓**	NR	**✓**	**✓**	**✓**	NR	NR	5 (62.50)
Lorite et al, 2007	NR	**✓**	**✓**	NR	NR	**✓**	**✓**	**✓**	5 (62.50)
Lu et al, 2019	NR	**✓**	NR	**✓**	**✓**	**✓**	NR	NR	4 (50.00)
Ma et al, 2009	NR	**✓**	NR	**✓**	**✓**	**✓**	NR	NR	4 (50.00)
Malhotra et al, 2017	NR	NR	NR	NR	NR	NR	NR	NR	0 (0.00)
Meng et al, 2022	NR	**✓**	**✓**	NR	NR	**✓**	**✓**	**✓**	5 (62.50)
Michel et al, 2020	NR	NR	NR	**✓**	**✓**	**✓**	**✓**	NR	4 (50.00)
Mierzchala et al, 2020	NR	**✓**	**✓**	NR	NR	**✓**	**✓**	NR	4 (50.00)
Mu et al, 2012	**✓**	**✓**	NR	**✓**	**✓**	**✓**	**✓**	NR	6 (75.00)
Myint et al, 2007	NR	**✓**	**✓**	NR	NR	**✓**	**✓**	**✓**	5 (62.50)
Myint et al, 2007	**✓**	NR	NR	NR	NR	NR	NR	NR	1 (12.50)
Nakatsukasa et al, 2011	NR	**✓**	**✓**	NR	NR	**✓**	**✓**	**✓**	5 (62.50)
Naz et al, 2019	**✓**	**✓**	NR	NR	NR	**✓**	**✓**	**✓**	6 (75.00)
Ogawa et al, 2018	**✓**	**✓**	NR	NR	NR	**✓**	**✓**	**✓**	6 (75.00)
Oh et al, 2017	**✓**	**✓**	**✓**	**✓**	**✓**	**✓**	**✓**	NR	7 (87.50)
Ohashi et al, 2013	NR	**✓**	NR	NR	NR	**✓**	**✓**	NR	3 (37.50)
Onesti et al, 2019	NR	NR	NR	NR	NR	**✓**	NR	NR	1 (12.50)
Palabiyik et al, 2016	**✓**	**✓**	NR	NR	NR	**✓**	**✓**	**✓**	5 (62.50)
Panitz et al, 2021	**✓**	**✓**	NR	NR	NR	**✓**	**✓**	**✓**	5 (62.50)
Pertovaara et al, 2005	**✓**	**✓**	NR	NR	NR	**✓**	**✓**	**✓**	5 (62.50)
Primiano et al, 2020	NR	NR	NR	NR	NR	NR	NR	NR	0 (0.00)
Ren et al, 2011	**✓**	NR	NR	**✓**	**✓**	**✓**	NR	NR	4 (50.00)
Roca et al, 1999	**✓**	NR	**✓**	**✓**	NR	**✓**	**✓**	**✓**	6 (75.00)
Rodrigues et al, 2021	NR	NR	**✓**	**✓**	NR	NR	**✓**	NR	3 (37.50)
Ruoppolo et al, 2014	NR	NR	NR	NR	NR	NR	NR	NR	0 (0.00)
Saito et al, 1979	NR	NR	NR	NR	NR	NR	NR	NR	0 (0.00)
Saito et al, 2022	NR	**✓**	NR	**✓**	**✓**	NR	NR	NR	3 (37.50)
Sakaguchi et al, 2011	**✓**	**✓**	NR	**✓**	**✓**	**✓**	NR	NR	5 (62.50)
Schwieler et al, 2020	**✓**	**✓**	**✓**	**✓**	**✓**	**✓**	**✓**	NR	7 (87.50)
Shi et al, 2019	**✓**	NR	NR	NR	NR	NR	NR	NR	1 (12.50)
Smolenska et al, 2020	NR	NR	NR	NR	NR	NR	NR	NR	0 (0.00)
Sorgdrager et al, 2017	NR	NR	**✓**	**✓**	NR	**✓**	NR	NR	3 (37.50)
Sorgdrager et al, 2019	NR	NR	NR	NR	NR	NR	NR	NR	0 (0.00)
Souissi et al, 2022	NR	NR	NR	NR	NR	NR	NR	NR	0 (0.00)
Sousa et al, 2021	NR	**✓**	**✓**	**✓**	**✓**	**✓**	**✓**	NR	6 (75.00)
Sultana et al, 2012	**✓**	**✓**	**✓**	**✓**	**✓**	**✓**	**✓**	**✓**	8 (100)
Sun et al, 2021	NR	NR	NR	**✓**	**✓**	**✓**	**✓**	NR	4 (50.00)
Sun et al, 2020	NR	NR	NR	NR	NR	NR	NR	NR	0 (0.00)
Suzuki et al, 2012	**✓**	NR	NR	NR	NR	NR	NR	NR	1 (12.50)
Suzuki et al, 2010	NR	NR	NR	NR	**✓**	**✓**	**✓**	NR	3 (37.50)
Suzuki et al, 2011	NR	NR	NR	NR	**✓**	**✓**	**✓**	NR	3 (37.50)
Taherizadeh et al, 2020	NR	NR	NR	NR	NR	NR	NR	NR	0 (0.00)
Tcherkas et al, 2001	NR	NR	NR	NR	NR	NR	NR	NR	0 (0.00)
Tezcan et al, 2022	**✓**	NR	NR	NR	NR	NR	NR	NR	1 (12.50)
Tong et al, 2018	NR	**✓**	NR	**✓**	**✓**	NR	**✓**	NR	4 (50.00)
Trepci et al, 2021	NR	NR	NR	NR	NR	**✓**	**✓**	NR	2 (25.00)
Tuka et al, 2021	NR	**✓**	NR	**✓**	**✓**	**✓**	**✓**	**✓**	6 (75.00)
Uchikura et al, 2003	NR	NR	NR	**✓**	NR	**✓**	NR	NR	2 (25.00)
Valko et al, 2019	NR	**✓**	NR	**✓**	**✓**	**✓**	NR	NR	4 (50.00)
Van Faassen et al, 2019	**✓**	**✓**	**✓**	**✓**	**✓**	NR	**✓**	NR	6 (75.00)
Walser et al, 1993	NR	NR	NR	NR	NR	NR	NR	NR	0 (0.00)
Wang et al, 2019	NR	NR	NR	NR	NR	NR	NR	NR	0 (0.00)
Wang et al, 2018	NR	NR	NR	NR	NR	NR	NR	NR	0 (0.00)
Widner et al, 2000	NR	NR	NR	NR	NR	NR	NR	NR	0 (0.00)
Wu et al, 2022	NR	NR	NR	NR	NR	NR	NR	NR	0 (0.00)
Wu et al, 2020	NR	NR	NR	NR	NR	NR	NR	NR	0 (0.00)
Wu et al, 2018	NR	NR	NR	NR	NR	NR	NR	NR	0 (0.00)
Xu et al, 2012	NR	NR	NR	NR	NR	NR	NR	NR	0 (0.00)
Yan et al, 2017	NR	**✓**	**✓**	**✓**	**✓**	**✓**	**✓**	NR	6 (75.00)
Yao et al, 2010	NR	NR	NR	NR	NR	NR	NR	NR	0 (0.00)
Yilmaz et al, 2020	**✓**	NR	NR	**✓**	NR	NR	NR	NR	2 (25.00)
Yoshitake et al, 2007	**✓**	**✓**	NR	**✓**	**✓**	**✓**	NR	NR	5 (62.50)
Zhang et al, 2020	NR	NR	NR	NR	NR	NR	NR	NR	0 (0.00)
Zhao et al, 2011	**✓**	**✓**	**✓**	**✓**	**✓**	**✓**	NR	NR	6 (75.00)
Zhen et al, 2011	**✓**	**✓**	NR	**✓**	**✓**	**✓**	**✓**	NR	6 (75.00)
Zhou et al, 2022	NR	NR	NR	NR	NR	NR	NR	NR	0 (0.00)
Zhou et al, 2019	NR	NR	NR	NR	NR	NR	NR	NR	0 (0.00)
Total (%)	35 (29.17)	47 (39.17)	23 (19.17)	43 (35.83)	39 (32.50)	53 (44.17)	44 (36.67)	13 (10.83)	NA

Abbreviations: ✓, reported; CC, calibration curve; LOD, limit of detection; LOQ, limit of quantification; NA, not applicable; NR, not reported.

### Analysis of variance

All grand means, MOD means and region means for TRP and KYN are summarised in Table 4. Normative data was not able to be generated for certain variables due to small sample sizes and/or insufficient data. Variables to which this applies include: (a) KYN serum within the American region; (b) KYN serum as measured by FL; (b) KYN plasma as measured by FL; (c) TRP serum as measured by UV-Vis; (d) TRP plasma as measured by UV-Vis; (e) KYN serum as measured by ECD; and (f) KYN plasma as measured by ECD.

**Table 4.** Normative data generated including grand mean concentrations (across all MODs and regions), region mean concentrations for TRP and KYN across serum and plasma, and MOD mean concentrations. Values presented are mean μM ± SD.

Mean	TRP serum	TRP plasma	KYN serum	KYN plasma
Grand mean	60.52 (15.38)	51.45 (10.47)	1.96 (0.51)	1.82 (0.54)
By region				
America	49.66 (2.75)	34.68 (9.90)	NR	0.94 (0.24)
Asia	60.30 (8.69)	53.81 (9.95)	1.68 (0.43)	1.61 (0.34)
Australia	67.26 (11.19)	42.87 (8.51)	2.43 (0.59)	2.12 (0.52)
Europe	60.73 (10.66)	52.92 (12.06)	2.07 (0.52)	2.29 (0.73)
Middle East	54.01 (18.44)	34.82 (10.52)	1.56 (0.62)	1.53 (0.72)
By MOD				
MS	60.39 (19.24)	50.07 (16.65)	1.90 (0.73)	1.91 (0.87)
FL	63.63 (15.36)	59.26 (15.02)	NR	NR
UV-Vis	NR	NR	2.22 (0.69)	1.95 (0.54)
ECD	63.04 (20.23)	26.49 (14.17)	NR	NR

Abbreviations: ECD, electron capture dissociation; FL, fluorescence detection; KYN, kynurenine; MOD, mode of detection; MS, mass spectrometry; NR, not reported; TRP, tryptophan; UV-Vis, ultraviolet-visible spectroscopy.

**Figure 2.**
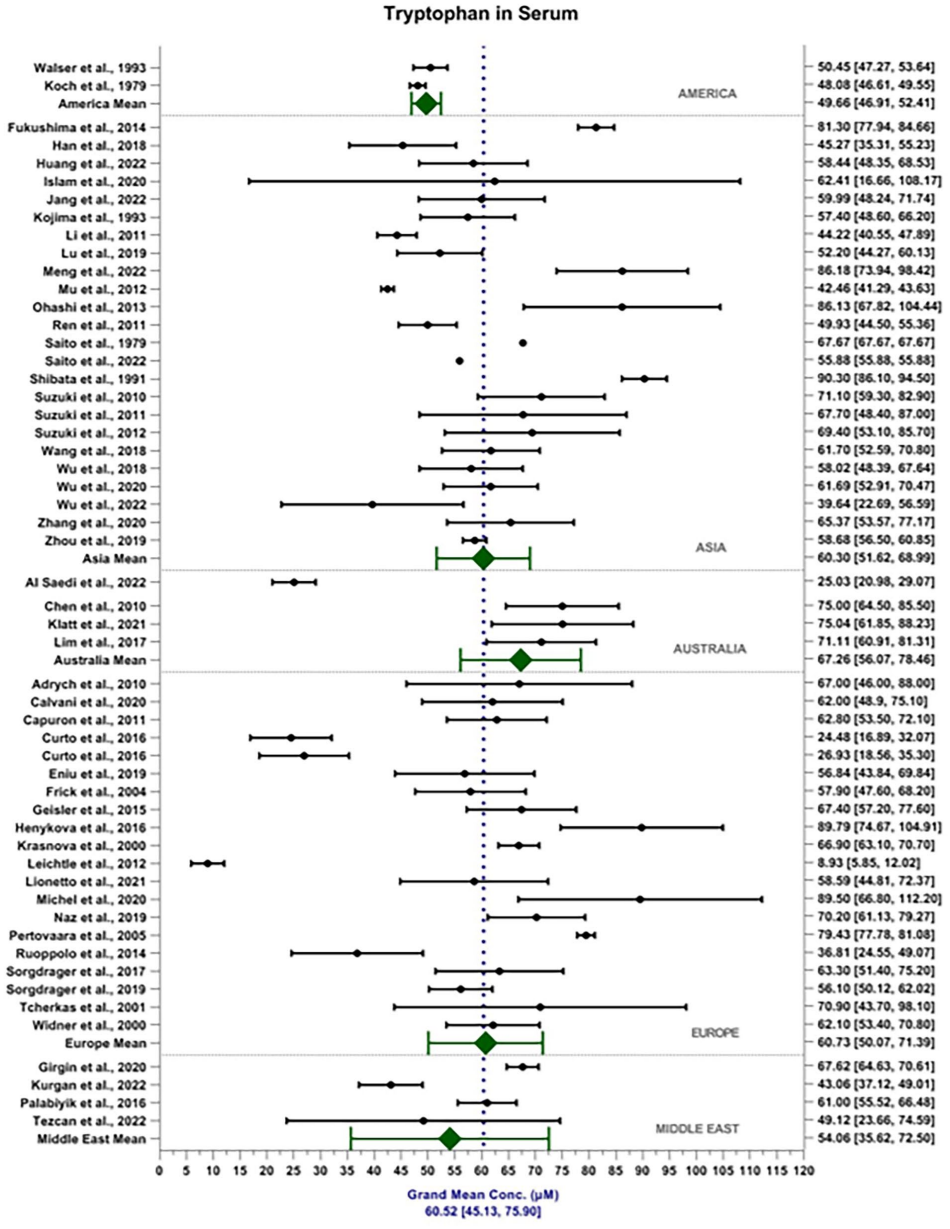
Forest plot depicting weighted grand mean concentrations [95%CI] of TRP in serum (μM) for each study together with region mean concentrations.

**Figure 3.**
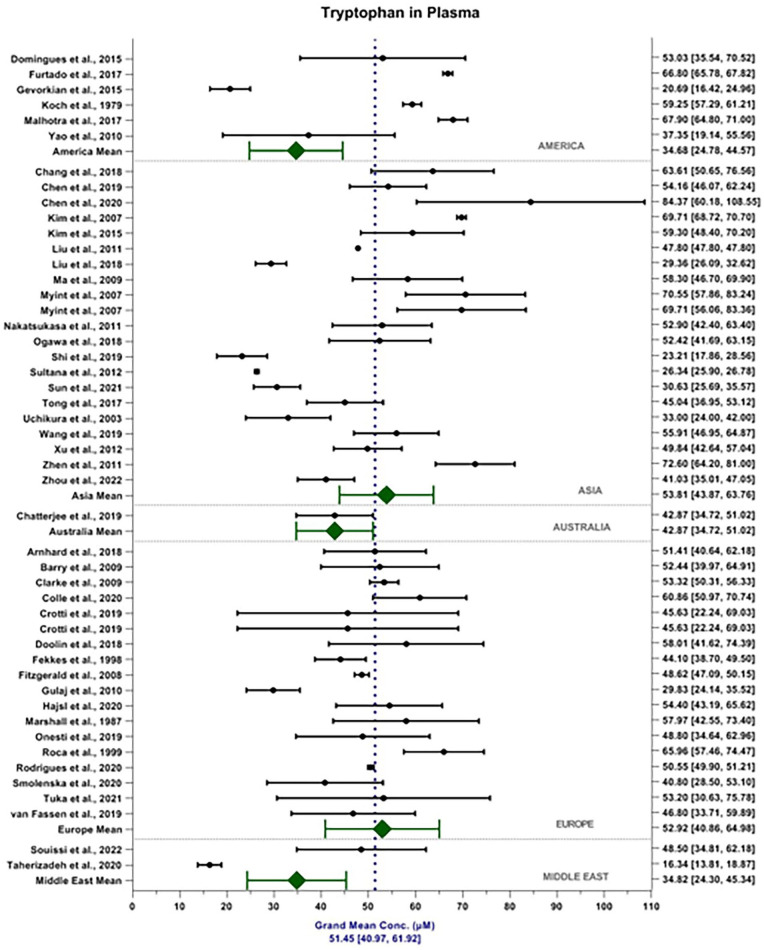
Forest plot depicting weighted grand mean concentrations [95%CI] of TRP in plasma (μM) for each study together with region mean concentrations.

The weighted grand mean concentration of TRP in serum across all MODs was calculated to be 60.52 ± 15.38 μM (see Figure 2). Mean reported TRP serum content in America, Asia, Australia, Europe and the Middle East were 49.66 ± 2.75, 60.30 ± 8.69, 67.26 ± 11.19, 60.73 ± 10.66 and 54.01 ±  18.44 μM, respectively. Mean reported TRP content detected in the serum by MS, FL and ECD were 60.39 ± 19.24, 63.63 ± 15.36 and 63.04 ± 20.23 μM, respectively. In comparison, the weighted grand mean concentration of TRP in plasma was calculated to be 51.45 ± 10.47 μm (Figure 3). Mean reported TRP plasma content in America, Asia, Australia, Europe and the Middle East were 34.68 ± 9.90, 53.81 ± 9.95, 42.87 ± 8.51, 52.92 ± 12.06 and 34.82 ±  10.52 μM, respectively. Mean reported TRP content detected in the plasma by MS, FL and ECD were 50.07 ± 16.65, 59.26 ± 15.02 and 26.49 ± 14.17 μM, respectively. MOD accounts for 2.96% of the total variance seen within the data, *F*(2, 6542) = 114.16, *P* < .001. Sample matrix accounted for 3.23% of the total variance seen within the data, *F*(1, 6542) = 248.93, *P* < .001. The interaction between MOD and sample matrix accounted for 1.53% of the total variance seen in the data *F*(2, 6542) = 58.94, *P* < .001.

**Figure 4.**
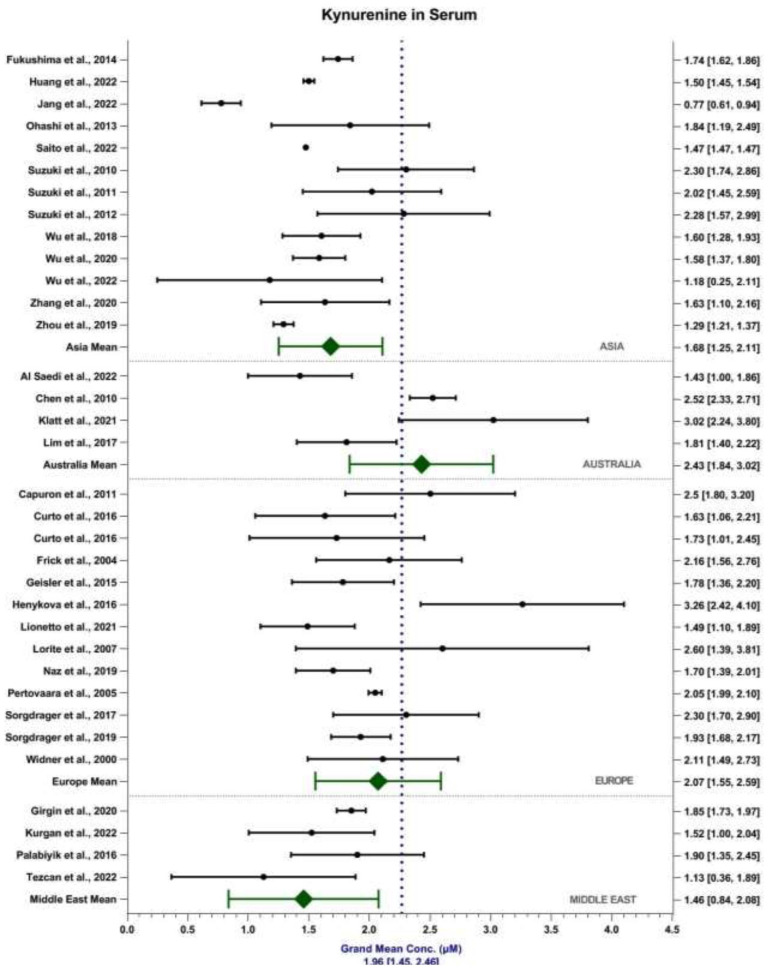
Forest plot depicting weighted grand mean concentrations [95%CI] of KYN in serum (μM) for each study together with region mean concentrations.

**Figure 5.**
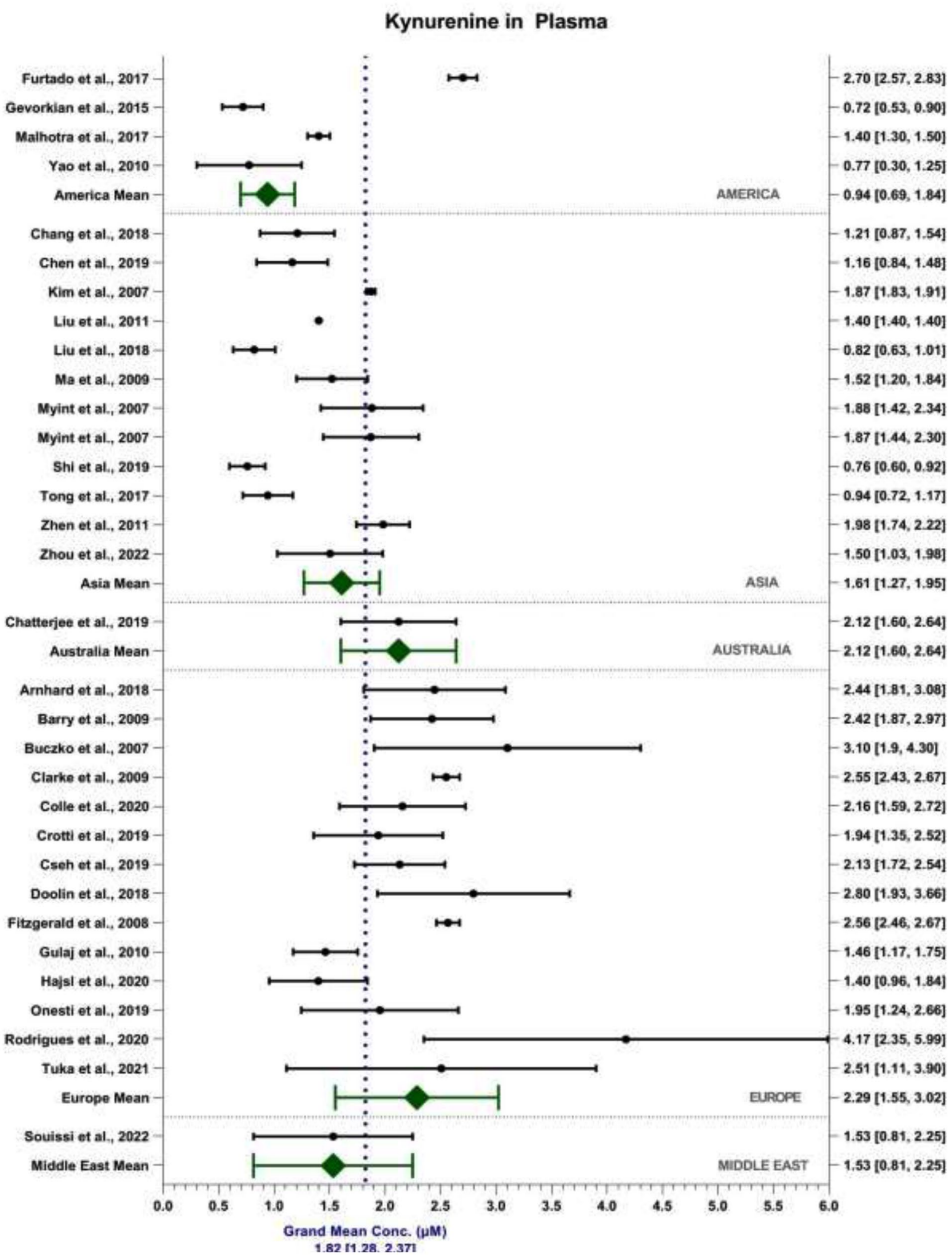
Forest plot depicting weighted grand mean concentrations [95%CI] of KYN in plasma (μM) for each study together with region mean concentrations.

The weighted grand mean concentration of KYN in serum across all MODs was calculated to be 1.96 ± 0.51 μM (Figure 4). Mean reported KYN serum content in Asia, Australia, Europe and the Middle East were 1.68 ± 0.43, 2.43 ± 0.59, 2.07 ± 0.52 and 1.56 ± 0.62 μM, respectively. Mean reported KYN content detected in the serum by MS and UV-Vis were 1.90 ± 0.73 μM and 2.22 ± 0.69 μM, respectively. In comparison, the weighted grand mean concentration of KYN in plasma across all MODs was calculated to be 1.82 ± 0.54 μM (Figure 5). Mean reported KYN plasma content in America, Asia, Australia, Europe and the Middle East were 0.94 ± 0.24, 1.61 ± 0.34, 2.12 ± 0.52, 2.29 ± 0.73 and 1.53 ± 0.72 μM, respectively. Mean reported KYN content detected in the plasma by MS and UV-Vis were 1.91 ± 0.87 μM and 1.95 ± 0.54 μM, respectively. MOD accounts for 1.30% of the total variance seen within the data, *F*(1, 3868) = 51.37, *P* < .001. Sample matrix accounted for 0.71% of the total variance seen within the data, *F*(1, 3868) = 27.96, *P* < .001. The interaction between MOD and sample matrix accounted for 0.79% of the total variance seen in the data, *F*(1, 3868) = 31.25, *P* < .001.

### Regression analyses

Outcomes from the adjusted weighted-variance OLS regressions including non-standardised β-coefficients and 95%CIs are listed in Table 5, for TRP and KYN concentrations in serum, plasma and blood, assessing predictors of biological sex, age and study year, with *P*-values for statistically significant outcomes bolded. In summary, female sex, age and study year were negatively correlated with TRP concentrations measured within the serum. Age and study year were also negatively correlated with TRP concentrations measured within the plasma, although no significant interaction was observed between biological sex and the concentrations measured. Across both serum and plasma, biological sex, age and study year were correlated with TRP concentrations measured within the serum. These trends were repeated when considering KYN, although age was positively correlated with KYN concentrations. Interestingly, biological sex was not correlated with both TRP and KYN concentrations within the plasma.

**Table 5.** Adjusted weighted-variance OLS regression analyses for TRP and KYN. TRP blood and KYN blood are the pooled sample sizes of serum and plasma studies.

	β-Coeff [95%CI]	*P*
TRP serum		
Proportion female*	−0.22 [−0.39, −0.05]	**.012**
Age	−0.20 [−0.38, −0.01]	**.036**
Study year	−1.37 [−1.67, −1.08]	**<.001**
TRP plasma		
Proportion female*	−0.03 [−0.08, 0.02]	.266
Age	−0.74 [−0.86, −0.61]	**<.001**
Study year	−1.58 [−1.87, −1.30]	**<.001**
TRP blood		
Proportion female*	−0.14 [−0.19, −0.09]	**<.001**
Age	−0.55 [−0.65, −0.45]	**<.001**
Study year	−1.35 [−1.55, −1.16]	**<.001**
KYN serum		
Proportion female*	−0.05 [−0.05, −0.04]	**<.001**
Age	0.01 [0.00, 0.01]	**.002**
Study year	−0.08 [−0.08, −0.07]	**<.001**
KYN plasma		
Proportion female*	−0.00 [−0.00, 0.00]	.431
Age	0.02 [0.01, 0.02]	**<.001**
Study year	−0.08 [−0.10, −0.07]	**<.001**
KYN blood		
Proportion female*	−0.02 [−0.02, −0.02]	**<.001**
Age	0.01 [0.01, 0.01]	**<.001**
Study year	−0.11 [−0.12, −0.11]	**<.001**

* Proportion female refers to the percentage of female participants as a proportion of the total sample size. This suggests that a positive β-Coeff value would indicate that as the proportion of female participants increases, metabolite concentrations increase and vice versa. A negative β-Coeff would therefore indicate higher metabolite concentrations in male participants.

Abbreviations: KYN, kynurenine; OLS, ordinary least squares; TRP, tryptophan.

### Non-blood sample matrices

All grand means for non-blood sample matrices are summarised in Table 6. TRP concentrations were reported in the urine (n = 15), CSF (n = 6), saliva (n = 2), tears (n = 1), faeces (n = 1) and whole blood (n = 1); while KYN concentrations were reported in the urine (n = 7), CSF (n = 6), saliva (n = 2) and faeces (n = 1).

**Table 6.** Normative data generated for TRP and KYN concentrations across urine, CSF, saliva, tears, faeces and whole blood. Values presented are mean μM ± SD.

Sample matrix	TRP	KYN
Urine	16.94 (20.40)	2.48 (4.41)
CSF	1.85 (0.22)	0.13 (0.03)
Saliva	0.77 (0.82)	0.05 (0.01)
Tears	17.10 (3.90)	NR
Faeces	0.23 (0.18)	0.00 (0.00)
Whole blood	32.32 (3.7)	NR

Abbreviations: CSF, cerebrospinal fluid; KYN, kynurenine; NR, not reported; TRP, tryptophan.

## Discussion

Precision medicine initiatives are currently limited by the availability and quality of the data summarising the KP profile.^143,144^ The current study addressed this gap by systematically mapping study characteristics according to MOD, metabolites studied, sample matrices and geographical location, and by calculating normative means of TRP and KYN concentrations. The methodologies and risk of bias reported within the literature were also critically appraised, the variance between MODs was determined based on the instrument reported, and the relationship between metabolite, biofluid, age, sex and study year were assessed.

FL and UV-Vis are suitable MODs for the analytical profiling of KP metabolites despite their less frequent use in the literature. The TRP and KYN metabolomic studies reported within this meta-analysis indicate that the most common MOD is MS (N = 80). High resolution MS is commonly used in lipidomics,^145^ proteomics^146^ and precision pharmacology.^147^ MS offers significant advantages in terms of sensitive analytical profiling,^148^ and FL and UV-Vis MODs are limited in their sensitivity by the light-emitting capacity of the metabolite.^149^ Nonetheless, the photophysical properties of TRP and KYN are well-characterised, and TRP and KYN are moderate emitters of intrinsic fluorescence and thus suitable for light-based detection.^150^ Moreover, concentrations of TRP and KYN within the serum, plasma and urine are mostly within the micromolar range.^24-26^ This suggests that FL and UV-Vis MODs, that are generally less favoured in terms of sensitivity, may be suitable cost-effective alternatives for these sample matrices.^148,149^ Studies aiming to quantify TRP and KYN in other sample matrices such as tears or saliva, where the concentration detected is generally lower,^87^ benefit from the sensitive analytical profiling of MS.^148^ Certain MOD averages reported within this meta-analysis, such as ECD, indicate extreme outliers. This may be due to the small number of studies reported within the literature, all of which vary in sample size. Further, the interaction between MOD and sample matrix accounted for 0.79% of the total variance, suggesting that choice of MOD should not affect the comparability of results between serum and plasma. Considering that the intra-coefficient and inter-coefficient of variation for a single assay will typically vary within an acceptable range of 3%-7% and the <1% variation between sample types, the interaction between sample type and MOD observed here at the population level is negligible.

TRP and KYN concentrations vary based on region, most likely due to genetic and dietary factors.^151^ Here, across serum and plasma TRP and KYN concentrations, American and Middle Eastern data were consistently lower than the grand population mean. It is generally well-understood that high protein and fat intake may increase the availability of TRP in the plasma and hence upregulate the KP, and vice versa.^151^ Differences in KP concentrations, including lower levels of TRP and KYN, may underpin heightened disease risk, including skeletal muscle atrophy,^152^ cognitive decline^152^ and higher mortality in cardiovascular disease,^153^ for example.

The sample matrices reported in this study suggest that there is a growing interest in minimally invasive sample types. The majority (66.67%) of the TRP urine papers have been published since 2015,^28,53,57,63,90,96,110,124,134,136^ while all included TRP saliva studies were published within this timeframe.^38,71^ Saliva metabolomics is an emerging field of research due to the diversity of metabolites present and the relative ease in sampling including decentralised approaches.^154^ Interest in saliva metabolomic profiling has also piqued due to reductions in the LOD of MS technologies and thus the improving analytical sensitivity.^148^ Urine is of interest in the field of biomarker discovery research as it is non-invasive, readily obtained in large quantities, and generally has high patient compliance.^155^ Urine may provide a relatively richer matrix for analysis, given that concentrations of metabolites are often higher in the urine (due to their excretion) when compared to serum.^156^ This indicates that urine may be more susceptible to metabolic changes when compared to the serum.^156^ Saliva and urine therefore present rich matrices for simple disease diagnostics in a minimally invasive and generally inexpensive manner.^154^ However, the relevance of these peripheral sample types in specific diseases warrants further investigation. Recent literature has established a relationship between cognitive function and gut dysbiosis,^157^ suggesting that peripheral-based biomarkers may be relevant sample matrices with respect to neurodegenerative diseases. The relationship between peripheral and central KP markers for neurodegeneration is supported by studies showing that plasma concentrations of KP metabolites KYN, 3-HAA, AA and PA are significantly correlated with their respective CSF levels.^158^

There are significant limitations in the accuracy and sensitivity of KP metabolomics approaches. Overall, 80.83% (N = 73) of studies reported at least one parameter of their independent methodological validation, chosen in compliance with the guidelines for pharmaceutical and biotechnology industries.^18^ Despite the dedicated experimental designs for robustness testing of LC-MS instruments reported within the literature,^159^ this parameter was only reported in 10.83% of the included studies.

The risk of bias appraisal questioned the reliability of the results reported here. Overall, only 51.67% of studies included in this meta-analysis were subjectively appraised to be of high quality. This was largely linked to poor reporting of methodological conditions (only reported in 34.17% of included studies). It is generally acknowledged that MS data presents various complexities linked to the various independently developed acquisition methods, diverse workflows, and non-standardised manual curation.^160^ The studies which were appraised to be of low quality often reported significant deviations from the calculated grand population mean.^43,60,69,84,130^ Nonetheless, this meta-analysis featured a very large sample size and TRP and KYN means are generally considered statistically reliable.

TRP and KYN concentrations were lower in female than male participants across both serum and plasma, an observation typically observed in younger healthy cohorts.^54^ With advancing age, TRP concentrations decreased, reflecting increased degradation of TRP in older age,^50^ while KYN concentrations increased. Recent studies conducted in older adults identified the same pattern of results,^161,162^ indicating the close link between age and metabolic activity related to TRP and the KP. Findings may help with the accurate profiling of the KP metabolome in diseases which show sex and age specific differences, such as AD.^163^ The decrease in TRP concentrations and elevated KYN concentrations suggest increased KP activation with advanced age. Upregulation of the KP may be linked to oxidative stress and neuroinflammation, pathologies linked to ageing.^164^

Strengths of this study include a broad eligibility criteria and thorough search strategy that detected ample records; the inclusion of only normative data; the inclusion of only targeted metabolomics data to ensure that precise metabolites have been profiled; and rigorous appraisal of the risk of bias and the validation parameters applied for methodological appraisal. Furthermore, this study had a large sample size (n = 8089) of relatively younger (⩽50 years) adults (47.35 ± 15.65), suggesting that it is statistically powered to detect accurate results. Limitations include an unequal distribution of sexes, with 77.67% of participants being of female biological sex. Moreover, meta-analyses were limited to serum and plasma data due to insufficient sample size in other minimally invasive sample types. This study was also limited to only TRP and KYN concentrations, and future studies should seek to assess other biologically active KP metabolites. Majority of the normative data presented in the literature is investigated within cohorts that are deemed healthy as screened against a singular health concern. This suggests that there may be some ambiguities in classifying normative data within the literature. Nonetheless, this review was intended to inform future empirical research and method development studies and successfully synthesised pilot data.

Future multi-site studies should seek to investigate and control for the relationship between geographical location and metabolite concentration, as well as clarify the relationship between study age and findings. Normative data should also be grouped into age and biological sex-specific brackets to ensure accuracy and specificity when being used for biomarker purposes. Specifically, studies should seek to report the metabolic profile of males and females separately. Moreover, future method development studies should seek to standardise acquisition methods, workflows and manual curation methods to improve the comparability of methodologies before moving into clinical and at-risk cohorts. Finally, future studies should consider comprehensively profiling the full KP metabolome within an array of biological sample matrices and should seek to identify any correlations between samples to determine the diagnostic accuracy of the KP across a range of inflammatory health conditions.

## Supplemental Material

sj-docx-1-try-10.1177_11786469231211184 – Supplemental material for Normative Data on Serum and Plasma Tryptophan and Kynurenine Concentrations from 8089 Individuals Across 120 Studies: A Systematic Review and Meta-Analysis Click here for additional data file. Supplemental material, sj-docx-1-try-10.1177_11786469231211184 for Normative Data on Serum and Plasma Tryptophan and Kynurenine Concentrations from 8089 Individuals Across 120 Studies: A Systematic Review and Meta-Analysis by Najwa-Joelle Metri, Ali S Butt, Ava Murali, Genevieve Z Steiner-Lim and Chai K Lim in International Journal of Tryptophan Research

sj-docx-2-try-10.1177_11786469231211184 – Supplemental material for Normative Data on Serum and Plasma Tryptophan and Kynurenine Concentrations from 8089 Individuals Across 120 Studies: A Systematic Review and Meta-Analysis Click here for additional data file. Supplemental material, sj-docx-2-try-10.1177_11786469231211184 for Normative Data on Serum and Plasma Tryptophan and Kynurenine Concentrations from 8089 Individuals Across 120 Studies: A Systematic Review and Meta-Analysis by Najwa-Joelle Metri, Ali S Butt, Ava Murali, Genevieve Z Steiner-Lim and Chai K Lim in International Journal of Tryptophan Research
