# Supplementary material for: Normative Data on Serum and Plasma Tryptophan and Kynurenine Concentrations from 8089 Individuals Across 120 Studies: A Systematic Review and Meta-Analysis
Source: Int J Tryptophan Res. 2023 Nov 29;16:11786469231211184. doi: 10.1177/11786469231211184 (PMC10687991; doi:10.1177/11786469231211184)
Supplement: sj-docx-1-try-10.1177_11786469231211184 – Supplemental material for Normative Data on Serum and Plasma Tryptophan and Kynurenine Concentrations from 8089 Individuals Across 120 Studies: A Systematic Review and Meta-Analysis [file sj-docx-1-try-10.1177_11786469231211184.docx]

1. **Supplementary Materials**

**Table S1.** Critical appraisal checklist adapted from the Joanna Briggs Institute Critical Appraisal Checklist for Analytical Cross-Sectional Studies (JBI), the Quality Assessment of Diagnostic Accuracy Assessment (QUADAS), and the QUADOMICS Tool.

| **Item No.** | **Tool** | **Item** | **Description** |
| --- | --- | --- | --- |
| 1 | JBI | Were the criteria for inclusion in the sample clearly defined? | The authors should provide clear inclusion and exclusion criteria that they developed prior to the recruitment of the study participants. The inclusion and exclusion criteria should be specified with sufficient detail and all the necessary information critical to the study. In the instance of healthy cohorts, the general definition of ‘healthy’ and the screening tools used to determine this should be described e.g., cognitively healthy older adults, metabolically healthy younger adults. |
| 2 | JBI | Were the study subjects and the setting described in detail? | The study sample should be described in sufficient detail so that other researchers can determine if it is comparable to the population of interest to them. The authors should provide a clear description of the population from which the study participants were selected or recruited, including demographics, location, and period. In the instance of healthy cohorts, the authors should describe the age, biological sex, and geographical location of the cohort. |
| 3 | QUADOMICS | Was the type of sample used fully described? | To score positively in this item, the report should present a details description of the type of sample e.g., serum, plasma, urine, saliva, etc. Moreover, the authors should specifically list the type of plasma specimen (e.g., EDTA, heparin, citrate) or urine specimen (e.g., midstream urine). Clinical and physiological factors should also be described e.g., fasting status. |
| 4 | QUADOMICS | Were the handling of specimens and pre-analytical procedures reported in sufficient detail and similar for the whole sample? | To score positively on this item, the study should describe any process related to the pre-analytical handling of the samples that could affect the results (e.g., number of freezing cycles, timing and storage of specimens, time from blood draw until centrifugation and storage, details on centrifugation conditions). |
| 5 | QUADAS, QUADOMICS | Was the execution of the index test described in sufficient detail to permit replication of the test? | The assay used to determine the concentrations of the metabolites in the samples should be described thoroughly. A citation to a technical article is not considered sufficient detail. In terms of mass spectrometry, description of the use of particular technologies (e.g., column chromatography, capillary electrophoresis) should be outlined. Analytical variability of the test should be described and controlled. The authors should explicitly describe the degree of instrument or observer variation and the methods used to control this variation e.g., validation parameters such as sensitivity, specificity, and calibration curves. A chromatogram should be provided. |
| 6 | QUADAS | Were uninterpretable or intermediate test results reported? | It is important that uninterpretable results are reported so that the impact of these results on test performance can be determined. If it is clear that all test results, including uninterpretable or indeterminate results, are reported then this item should be scored positively. |
| JBI = Joanna Briggs Institute Critical Appraisal Checklist for Analytical Cross-Sectional Studies; QUADAS = Quality Assessment of Diagnostic Accuracy Assessment. | | | |

**Table S2.** Study characteristics of included studies including the study type, mode of detection (MOD), country, sample size, sex, and age, grouped by specimen type.

| **Citation** | **Study Type** | **MOD** | **Country** | **Biospecimen** | **Population Size** | **Population Sex** | **Mean Age (SD)** |
| --- | --- | --- | --- | --- | --- | --- | --- |
| Chen et al., 2010* | Clinical | UV-Vis + FL | Australia | CSF | 35 | NR | 35.80 (3.00) |
| Rodrigues et al., 2021* | Clinical | MS | UK | CSF | 20 | NR | NR |
| Schwieler et al., 2020 | Clinical | MS | Sweden | CSF | 13 | 5M, 8F | 40.40 (14.60) |
| Sorgdrager et al., 2019* | Clinical | MS | Belgium | CSF | 39 | 18M, 21F | 71.30 (10.70) |
| Trepci et al., 2021 | Clinical | MS | Sweden | CSF | 80 | 39M, 41F | NR |
| Sun et al., 2020 | Clinical | MS | China | Faeces | 38 | 24M, 14F | 56.85 (10.99) |
| Arnhard et al., 2018 | Analytical | MS | Austria | Plasma | 100 | NR | NR |
| Barry et al., 2009 | Clinical | FL + PDA | Ireland | Plasma | 36 | 26M, 10F | 33.70 (6.60) |
| Buczko et al., 2007* | Clinical | UV-Vis | Poland | Plasma | 19 | 9M, 10F | 61.00 (15.00) |
| Chang et al., 2018 | Clinical | MS | Taiwan | Plasma | 82 | 40M, 32F | 62.83 (12.66) |
| Chatterjee et al., 2019 | Clinical | FL | Australia | Plasma | 65 | 19M, 46F | 77.61 (5.55) |
| Chen et al., 2019 | Analytical | MS | China | Plasma | 100 | NR | NR |
| Chen et al., 2020 | Clinical | MS | China | Plasma | 75 | 32M, 43F | 37.19 (9.66) |
| Clarke et al., 2009 | Clinical | UV-VIS + FL | Ireland | Plasma | 26 | 26M, 0F | 32.20 |
| Colle et al., 2020 | Clinical | MS | France | Plasma | 214 | 90M, 124F | 45.50 (NR) |
| Crotti et al., 2019 | Clinical | UV-VIS + FL | Italy | Plasma | 5 | NR | NR |
| Cseh et al., 2019 | Analytical | MS | Hungary | Plasma | 8 | NR | NR |
| Domingues et al., 2015 | Clinical | MS | Brazil | Plasma | 38 | NR | NR |
| Doolin et al., 2018 | Clinical | MS | Ireland | Plasma | 37 | 18M, 19F | 80.86 (10.78) |
| Fekkes et al., 1998 | Clinical | FL | Netherlands | Plasma | 17 | 17M, 0F | 70.10 (1.30) |
| Fitzgerald et al., 2008 | Clinical | FL | Ireland | Plasma | 33 | 0M, 33F | 41.30 (12.80) |
| Furtado et al., 2017 | Clinical | MS | Brazil | Plasma | 18 | 7M, 11F | NR |
| Gevorkian et al., 2015 | Clinical | ECD | USA | Plasma | 140 | 72M, 68F | 50.80 (8.80) |
| Gulaj et al., 2010 | Clinical | UV-Vis + FL | Poland | Plasma | 18 | 5M, 13F | 76.17 (7.30) |
| Hajsl et al., 2020 | Clinical | MS | Czech Republic | Plasma | 25 | 14M, 11F | NR |
| Kim et al., 2015 | Clinical | MS | South Korea | Plasma | 70 | 44M, 26F | 63.20 (8.90) |
| Kim et al., 2009 | Clinical | UV-Vis + FL | South Korea | Plasma | 174 | 78M, 96F | 32.49 (10.69) |
| Koch et al., 1979 | Analytical | ECD | USA | Plasma | 11 | NR | NR |
| Liu et al., 2018 | Analytical | ECD | China | Plasma | NR | NR | NR |
| Ma et al., 2009 | Clinical | UV | China | Plasma | 10 | NR | NR |
| Malhotra et al., 2017 | Clinical | MS | USA | Plasma | 10 | 4M, 6F | 44.00 (12.00) |
| Myint et al., 2007 | Clinical | UV-Vis + FL | Korea | Plasma | 80 | 40M, 40F | 39.06 (8.75) |
| Myint et al., 2007 | Clinical | UV-Vis + FL | Korea | Plasma | 189 | 76M, 113F | 32.49 (10.69) |
| Nakatsukasa et al., 2011 | Clinical | MS | Japan | Plasma | 34 | 17M, 17F | NR |
| Ogawa et al., 2018 | Clinical | FL | Japan | Plasma | 217 | 100M, 117F | 41.20 (13.90) |
| Onesti et al., 2019 | Clinical | MS | Belgium | Plasma | 146 | 0M, 146F | NR |
| Roca et al., 1999 | Clinical | UV-Vis | Spain | Plasma | 29 | 13M, 16F | NR |
| Rodrigues et al., 2021* | Clinical | MS | UK | Plasma | 20 | NR | NR |
| Shi et al., 2019 | Clinical | MS | China | Plasma | 11 | 8M, 3F | 51.09 (10.77) |
| Smolenska et al., 2020 | Clinical | MS | Poland | Plasma | 27 | NR | NR |
| Souissi et al., 2022 | Clinical | UV-Vis | Tunisia | Plasma | 50 | NR | NR |
| Sultana et al., 2012 | Analytical | UV-Vis | Pakistan | Plasma | 10 | 10M, 0F | NR |
| Sun et al., 2021 | Clinical | MS | China | Plasma | 401 | 163M, 238F | 53.15 (6.64) |
| Taherizadeh et al., 2020 | Clinical | MS | Iran | Plasma | 37 | 20M, 17F | 64.24 (13.08) |
| Tong et al., 2018 | Clinical | MS | China | Plasma | 18 | NR | NR |
| Tuka et al., 2021 | Clinical | MS | Hungary | Plasma | 34 | 0M, 34F | 30.50 (12.77) |
| Uchikura et al., 2003 | Analytical | ECL | Japan | Plasma | 10 | NR | NR |
| Van Faassen et al., 2019 | Clinical | MS | Netherlands | Plasma | 68 | 35M, 33F | NR |
| Wang et al., 2019 | Analytical | MS | China | Plasma | 475 | 189M, 286F | 58.67 (6.30) |
| Xu et al., 2012 | Clinical | MS | China | Plasma | 25 | 9M, 16F | 32.12 (8.15) |
| Yao et al., 2010 | Clinical | ECD | USA | Plasma | 30 | 18M, 12F | NR |
| Zhen et al., 2011 | Clinical | UV-Vis | China | Plasma | 20 | 10M, 10F | NR |
| Zhou et al., 2022 | Clinical | MS | China | Plasma | 60 | 30M, 30F | 30.10 (11.50) |
| Buczko et al., 2007 | Clinical | UV-Vis | Poland | Saliva | 19 | 9M, 10F | 61.00 (15.00) |
| Cheng et al., 2015 | Clinical | MS | China | Saliva | 28 | 0M, 28F | NR |
| Kurgan et al., 2022 | Clinical | MS | Turkey | Saliva | 20 | 8M, 12F | 39.60 (6.70) |
| Adrych et al., 2010 | Clinical | MS | Poland | Serum | 21 | 21M, 0F | 34.00 (13.00) |
| Al Saedi et al., 2022 | Clinical | UV-Vis + FL | Australia | Serum | 28 | 4M, 24F | NR |
| Calvani et al., 2020 | Clinical | MS | Spain, France | Serum | 30 | 14M, 16F | 74.60 (4.30) |
| Capuron et al., 2011 | Analytical | UV-Vis + FL | France | Serum | 284 | 87M, 197F | 79.90 (4.50) |
| Chen et al., 2010* | Clinical | UV-Vis + FL | Australia | Serum | 35 | NR | 35.80 (3.00) |
| Curto et al., 2016 | Clinical | MS | Italy | Serum | 35 | 27M, 8F | 44.80 (7.64) |
| Curto et al., 2016 | Clinical | MS | Italy | Serum | 84 | 15M, 69F | 40.40 (9.43) |
| Eniu et al., 2019 | Clinical | MS | Romania | Serum | 26 | 0M, 26F | NR |
| Frick et al., 2004 | Analytical | UV-Vis + FL | Austria | Serum | 43 | 22M, 21F | 66.3 |
| Fukushima et al., 2014 | Clinical | MS | Japan | Serum | 27 | 12M, 15F | 26.50 (5.60) |
| Geisler et al., 2015 | Clinical | UV-Vis + FL | Austria | Serum | 100 | 58M, 42F | 49.00 (11.40) |
| Girgin et al., 2020 | Clinical | UV-Vis + FL | Turkey | Serum | 30 | 20M, 10F | 37.0 (1.30) |
| Han et al., 2018 | Clinical | MS | China | Serum | 30 | 21M, 9F | NR |
| Henykova et al., 2016 | Analytical | MS | Czech Republic | Serum | 18 | 4M, 14F | NR |
| Huang et al., 2022 | Clinical | MS | China | Serum | 62 | 36M, 26F | 43.40 (1.30) |
| Islam et al., 2020 | Clinical | UV-Vis | Bangladesh | Serum | 248 | 102M, 146F | NR |
| Jang et al., 2022 | Clinical | MS | South Korea | Serum | 35 | 32M, 3F | 24.66 (2.99) |
| Klatt et al., 2021 | Clinical | MS | Australia | Serum | 93 | 49M, 44F | NR |
| Koch et al., 1979* | Analytical | ECD | USA | Serum | 11 | NR | NR |
| Krasnova et al., 2000 | Clinical | ECD | Russia | Serum | 10 | NR | NR |
| Leichtle et al., 2012 | Clinical | MS | Germany | Serum | 58 | 26M, 32F | NR |
| Li et al., 2011 | Clinical | FL | China | Serum | 100 | 52M, 48F | NR |
| Lim et al., 2017 | Clinical | MS | Australia | Serum | 49 | 14M, 35F | 45.29 (11.70) |
| Lionetto et al., 2021 | Clinical | MS | Italy | Serum | 239 | 87M, 152F | NR |
| Lorite et al., 2007 | Clinical | UV-Vis + FL | Spain | Serum | 5 | NR | NR |
| Lu et al., 2019 | Clinical | MS | Singapore | Serum | 76 | NR | NR |
| Meng et al., 2022 | Clinical | MS | China | Serum | 96 | 96M, 0F | 78.01 (6.99) |
| Michel et al., 2020 | Clinical | MS | Germany | Serum | 20 | 13M, 7F | NR |
| Mierzchala et al., 2020 | Clinical | MS | Poland | Serum | 30 | NR | NR |
| Mu et al., 2012 | Clinical | FL | China | Serum | 110 | 58M, 52F | NR |
| Naz et al., 2019 | Clinical | MS | Sweden | Serum | 39 | 19M, 20F | NR |
| Ohashi et al., 2013 | Analytical | MS | Japan | Serum | 19 | 8M, 11F | 23.60 (3.50) |
| Palabiyik et al., 2016 | Clinical | UV-Vis + FL | Turkey | Serum | 30 | 13M, 17F | 36.00 (2.00) |
| Panitz et al., 2021 | Clinical | MS | Germany | Serum | 43 | 19F, 24M | NR |
| Pertovaara et al., 2005 | Clinical | UV-Vis + FL | Finland | Serum | 309 | 170M, 139F | 45.00 (11.00) |
| Ren et al., 2011 | Clinical | FL | China | Serum | 120 | 62M, 58F | NR |
| Ruoppolo et al, 2014 | Clinical | MS | Italy | Serum | 76 | 35M, 41F | NR |
| Saito et al., 1979 | Clinical | UV-Vis + FL | Japan | Serum | 8 | NR | NR |
| Saito et al., 2022 | Clinical | MS | Japan | Serum | 59 | 29M, 30F | 52.50 (7.93) |
| Sorgdrager et al., 2017 | Clinical | MS | Netherlands | Serum | 406 | 163M, 243F | 42.90 (14.70) |
| Sorgdrager et al., 2019* | Clinical | MS | Belgium | Serum | 39 | 18M, 21F | 71.30 (10.70) |
| Suzuki et al., 2012 | Clinical | MS | Japan | Serum | 85 | 48M, 37F | NR |
| Suzuki et al., 2010 | Clinical | MS | Japan | Serum | 45 | 34M, 11F | 63.40 (9.40) |
| Suzuki et al., 2011 | Clinical | MS | Japan | Serum | 64 | 39M, 25F | NR |
| Tcherkas et al., 2001 | Clinical | ECD | Russia | Serum | 16 | NR | NR |
| Tezcan et al., 2022 | Clinical | MS | Turkey | Serum | 80 | 38M, 42F | 35.09 (7.09) |
| Walser et al, 1993 | Clinical | MS | USA | Serum | 22 | 12M, 10F | NR |
| Wang et al., 2018 | Clinical | MS | China | Serum | 298 | NR | 62.00 (17.00) |
| Widner et al., 2000 | Clinical | MS | Austria | Serum | 20 | 10M, 10F | NR |
| Wu et al., 2022 | Clinical | MS | China | Serum | 10 | NR | NR |
| Wu et al., 2020 | Clinical | MS | China | Serum | 36 | 6M, 30F | 65.83 (7.30) |
| Wu et al., 2018 | Clinical | MS | China | Serum | 135 | 42M, 93F | 66.99 (6.77) |
| Zhang et al., 2020 | Clinical | MS | China | Serum | 79 | 42M, 37F | 28.85 (9.43) |
| Zhou et al., 2019 | Clinical | MS | China | Serum | 72 | 41M, 31F | 36.30 (11.90) |
| Nakatsukasa et al., 2011* | Clinical | MS | Japan | Tears | 34 | 17M, 17F | NR |
| Bai et al., 2021 | Clinical | ELISA | China | Urine | 41 | 23M, 18F | 66.83 (7.85) |
| Bassi et al., 2017 | Clinical | MS | USA | Urine | 10 | NR | NR |
| Bizzarri et al., 1990 | Clinical | UV-Vis | Italy | Urine | 9 | NR | NR |
| Galla et al., 2021 | Analytical | MS | Hungary | Urine | 10 | NR | NR |
| Gomez-Gomez et al., 2017 | Clinical | MS | Spain | Urine | 25 | 2M, 23F | NR |
| Huang et al., 2021 | Clinical | MS | China | Urine | 40 | NR | NR |
| Koch et al., 1979* | Analytical | ECD | USA | Urine | 11 | NR | NR |
| Oh et al., 2017 | Clinical | MS | South Korea | Urine | 163 | NR | 50.92 (15.39) |
| Primiano et al., 2020 | Clinical | MS | Italy | Urine | 12 | 4M, 8F | NR |
| Sakaguchi et al., 2011 | Analytical | FL | Japan | Urine | 7 | 7M, 0F | NR |
| Sousa et al., 2021 | Clinical | UV-Vis + FL | Portugal | Urine | 6 | 6M, 0F | NR |
| Valko et al., 2019 | Clinical | FL | Slovakia | Urine | 51 | 35M, 16F | 36.60 (10.90) |
| Yan et al., 2017 | Analytical | MS | Australia | Urine | 10 | 6M, 4F | NR |
| Yilmaz et al., 2020 | Clinical | MS | USA | Urine | 29 | 13M, 16F | 79.12 (6.28) |
| Yoshitake et al., 2007 | Analytical | FL | Japan | Urine | 7 | NR | NR |
| Zhao et al., 2011 | Analytical | UV-Vis + FL | China | Urine | NR | NR | NR |
| *Note.* ECD: Electron Capture Detector; ECL: Electrochemiluminescence; FL: Fluorescence Detection; MOD: Mode of Detection; MS: Mass Spectrometry; SD: Standard Deviation; UV-Vis: Ultraviolet-Visible Spectroscopy. | | | | | | | |

**Table S3.** Chromatographic conditions of the included studies, including column phase and diameter, particle size, mobile phases, flow rate, injection volume, column temperature, program time, and detection wavelength (where applicable), grouped by MOD.

| **Citation** | **MOD** | **Column Phase** | **Column Diameter (mm)** | **Particle Size**  **(µM)** | **Mobile Phase A** | **Mobile Phase B** | **Flow Rate**  **(mL/min)** | **Injection Volume (µL)** | **Column Temp. (°C)** | **Detection Wavelength (nm)** | **Program Time (min)** |
| --- | --- | --- | --- | --- | --- | --- | --- | --- | --- | --- | --- |
| Gevorkian et al., 2015 | ECD | C_18_ | 250 × 4.6 | 5 | NR | NR | 0.7 – 1.2 | NR | 35 | NA | NR |
| Koch et al., 1979 | ECD | C_18_ | 150 × 4.0 | 10 | 0.5M AA, 15 % MeOh | McIlvaine buffer, 20 % MeOh | 1 | 20 | NR | NA | NR |
| Krasnova et al., 2000 | ECD | C_18_ | 250 × 4.0 | 5 | MeOh, Sodium Hydrophosphate, Sodium Dihydrophosphate, 0.002M EDTA | NR | 0.7 | 20 | NR | NA | NR |
| Liu et al., 2018 | ECD | C_18_ | 250 × 4.6 | 4 | Acetate Buffer, MeOh (4:1, v/v) | NR | 1 | 20 | NR | NA | NR |
| Tcherkas et al., 2001 | ECD | C_18_ | 250 × 4.0 | 5 | 0.01 – 0.02M Sodium Dihydrogenphosphate, 0.01 – 0.02 Disodium Hydrogenphosphate, 10 % - 19 % MeOh (v/v), 2mM Na2EDTA | NR | 0.7 | 20 | 26 | NA | 60 |
| Yao et al., 2010 | ECD | C_18_ | 250 × 4.6 | 5 | 10.3gL-1 Sodium Pentane Sulfonate, 5mL-1 Acetic Acid | MeOh/ACN/Isopropanol (8/1/1), 8gL-1 Lithium Acetate, 20mL-1 Acetic Acid | NR | NR | 35 | NA | NR |
| Uchikura et al., 2003 | ECL | C_18_ | 150 × 4.6 | NR | ACN, 10mM KH2PO4 | NR | 1 | 10 | 50 | NA | NR |
| Bai et al., 2021 | ELISA | NA | NA | NA | NA | NA | NA | NA | NA | NA | NA |
| Al Saedi et al., 2022* | FL | C_18_ | 150 × 2.1 | 1.8 | 0.2mM SA | NR | 0.75 | 20 | 38 | KYN: 365  TRP: Ex/Em 280/438 | 12 |
| Barry et al., 2009* | FL | C_18_ | 150 × 2.0 | 2 | 50mM Acetic Acid, 100mM ZA, 3 % (v/v ACN) | NR | 0.3 | NR | NR | FL: Ex/Em 254/404  PDA: 210 – 400 | NR |
| Capuron et al., 2011* | FL | C_18_ | 244 | 5 | 0.015mol/L Degassed PP (pH 6.4) | 27mL/L ACN | 0.8 | NR | 25 | KYN: 360  TRP: Ex/Em 285/365 | NR |
| Chatterjee et al., 2019 | FL | C_18_ | 100 × 2.1 | 1.8 | 50nM SA, 25mM ZA, 2.25 % ACN | 10 % ACN | 0.75 | NR | 38 | Ex/Em 344/388 | 10 |
| Chen et al., 2010* | FL | C_18_ | NR | NR | 0.1M AA (pH 4.65) | NR | 1 | 30 | 22 | KYN: 360  TRP: Ex/Em 285/365 | NR |
| Clarke et al., 2009* | FL | C_18_ | 150 × 2.0 | 2 | 50mM Acetic Acid, 100mM ZA, 3 % (v/v) ACN | NR | 0.3 | 20 | 30 | KYN: 330  TRP: Ex/EM 254/404 | 30 |
| Crotti et al., 2019* | FL | C_18_ | 250 × 4.6 | 5 | ACN, 0.005 Phosphate Buffer (15:85 v/v) | NR | 1 | NR | NR | KYN: 360  TRP: Ex/Em 285/345 | NR |
| Fekkes et al., 1998 | FL | C_18_ | 150 × 3.0 | 5 | NR | NR | 0.6 | NR | 28 | NR | NR |
| Fitzgerald et al., 2008 | FL | C_18_ | 150 × 2.0 | NR | 50mmol/L Acetic Acid, 100mL/L ZA, 3 % (v/v) ACN | NR | NR | NR | NR | Ex/Em 254/404 | NR |
| Frick et al., 2004* | FL | C_18_ | NR | NR | NR | NR | 0.9 | NR | NR | KYN: 360  TRP: Ex/Em 285/365 | NR |
| Geisler et al., 2015* | FL | C_18_ | NR | 5 | 15mmol/L Acetic Acid-SA (pH 4.0) | NR | NR | NR | NR | KYN: 360  TRP: Ex/Em 286/366 | NR |
| Girgin et al., 2020* | FL | NR | NR | NR | 15mM pH 6.5 Monopotassium Phosphate, 0.7 % ACN | NR | 0.8 | NR | NR | KYN: 360  TRP: Ex/Em 286/Em | NR |
| Gulaj et al., 2010* | FL | C_18_ | 150 × 2.1 | NR | KYN: 0.1M Acetic Acid, 0.1M AA, 2 % ACN  TRP: 50mM Acetic Acid, 0.25M ZA, 1.2 % ACN | NR | 0.2 | NR | NR | KYN: 365  TRP: Ex/Em 254/404 | NR |
| Kim et al., 2009* | FL | C_18_ | 100 × 4.7 | NR | TRP: 57.2g Na2HPO412H2O, 60mL ACN in water  KYN: 250mM ZA in water | TRP: 420 water/280 CAN/320 MeOh  KYN: 9 % ACN | NR | NR | NR | KYN: 365  TRP: Ex/Em 340/440 | NR |
| Li et al., 2011 | FL | C_18_ | 250 × 4.6 | 5 | 10 % ACN in water (v/v) | NR | 1 | 20 | NR | Ex/Em 285/353 | NR |
| Lorite et al., 2007* | FL | C_18_ | 244 × 4.0 | 5 | Phosphate | NR | 0.8 | 100 | NR | KYN: 360  TRP: Ex/Em 285/365 | NR |
| Mu et al., 2012 | FL | C_18_ | 150 × 4.6 | 5 | 0.1mol/L Monopotassium Phosphate, MeOh (85:15, v/v) | NR | 1 | 20 | 25 | Ex/Em 200 – 450/250 - 500 | 12 |
| Myint et al., 2007* | FL | C_18_ | 100 × 4.7 | 5 | 50mM ZA, Acetic Acid, 1.0 % ACN | NR | 1 | 100 | NR | KYN: 365  TRP: Ex/Em 340/440 | NR |
| Myint et al., 2007* | FL | C_18_ | 100 × 4.7 | NR | TRP: 57.2g NA2HPO412H2O, 160mL ACN in water  KYN: 250mM ZA in water | TRP: 420 water/280 ACN/320 MeOh | NR | NR | NR | KYN: 365  TRP: Ex/Em 340/440 | NR |
| Ogawa et al., 2018 | FL | C_18_ | 100 × 2.1 | NR | NR | NR | NR | NR | NR | NR | NR |
| Palabiyik et al., 2016* | FL | C_18_ | 250 × 4.6 | 5 | 15mM Phosphate, 27mL/L ACN | NR | 0.8 | 100 | NR | KYN: 360  TRP: Ex/Em 286/366 | NR |
| Pertovaara et al., 2005* | FL | C_18_ | 50 × 2.1 | 5 | 15mmol/L SA in Acetic Acid, 27mL/L ACN | NR | 0.9 | 10 | NR | KYN: 360  TRP: Ex/Em 266/366 | NR |
| Ren et al., 2011 | FL | C_18_ | 250 × 4.6 | 5 | 10 % ACN (v/v) | NR | 1.2 | 20 | 25 | Ex/Em 285/353 | NR |
| Saito et al., 1979* | FL | CK-10-S | 300 × 4.6 | NR | NR | NR | 0.73 | NR | 60 | KYN: 280  TRP: Ex/Em 280/340 | 60 |
| Sakaguchi et al., 2011 | FL | C_18_ | 150 × 4.6 | 5 | MeOh, water, TFA (2.5:97.5:0.05, v/v) | MeOh, water, TFA (60:40:0.05, v/v) | 1 | 20 | 30 | Ex/Em 280/320 | NR |
| Sousa et al., 2021* | FL | C_18_ | NR | 3 | 20mM AF in 0.01 % FA in water, ACN, Ethanol (95/2/3, v/v/v) | NR | 0.7 | 10 | 25 | KYN: 365  TRP: Ex/Em 280/348 | 27 |
| Valko et al., 2019 | FL | C_18_ | 250 × 4.0 | 5 | 15 % ACN | NR | 0.8 | 40 | 30 | Ex/Em 280 – 315/350 – 425 | 30 |
| Yoshitake et al., 2007 | FL | C_18_ | 250 × 4.6 | 5 | 0.04 % TFA/MeOh (99:1, v/v) | 0.04 % TFA/MeOh (90:10, v/v) | 1 | 20 | 21 - 25 | NR | 75 |
| Zhao et al., 2011* | FL | C_18_ | 250 × 4.6 | 5 | 20mmol/L NaAc, 30mmol/L HAc, 3 % MeOh | 20mmol/L NaAc/HAc, 10 % MeOh, 19 % ACN | 1 | 50 | 25 | KYN: 365  TRP: Ex/Em 292/340 | 30 |
| Adrych et al., 2010 | MS | C_18_ | 150 × 2.0 | 3 | 0 % to 60 % ACN | NR | 0.2 | NR | 275 | NA | 12 |
| Arnhard et al., 2018 | MS | NH_2_ | 150 × 1.0 | 3 | 50 % to 5 % ACN in AA | NR | 0.035 | 5 | 50 | NA | 28.5 |
| Bassi et al., 2017 | MS | NR | NR | NR | 5mM AA, Methanol | NR | NR | 20 | NR | NA | NR |
| Calvani et al., 2020 | MS | C_18_ | 150 × 2.1 | 1.6 | 0.1 % FA in water | 0.1 % ACN in FA | 0.5 | NR | NR | NA | 9 |
| Chang et al., 2018 | MS | C_18_ | 100 × 2.1 | 1.8 | 0.5 % FA in water | 0.5 % FA in ACN | 0.4 | NR | 30 | NA | NR |
| Chen et al., 2019 | MS | C_18_ | 100 × 3.0 | 1.8 | 0.1 % FA in water, v/v | 100 % ACN | 0.3 | 2 | 30 | NA | 5 |
| Chen et al., 2020 | MS | C_18_ | 100 × 2.1 | 1.7 | A: 0.1% FA in water | FA, ACN, water | 0.2 – 0.4 | 1 | 40 | NA | NR |
| Cheng et al., 2015 | MS | C_18_ | 100 × 2.1 | 1.7 | 10mM AF | ACN in water (95:5), 2mM AF | 0.2 | 10 | 45 | NA | NR |
| Colle et al., 2020 | MS | C_18_ | 100 × 2.1 | 1.7 | 0.1 % FA in water | 0.1 % FA in ACN | 0.5 | 5 | 40 | NA | NR |
| Cseh et al., 2019 | MS | C_18_ | 150 × 4.6 | 5 | 200mM ZA (pH 6.2) | NR | 1.2 | 20 | NR | KYN: 200 – 800  TRP: Ex/Em 300/495 | NR |
| Curto et al., 2016 | MS | PFP | 100 × 2.1 | 2.6 | 0.1 % FA in water | 100 % MeOH | 0.3 | 80 | 25 | NA | 16 |
| Curto et al., 2016 | MS | PFP | 100 × 2.1 | 2.6 | 0.1 % FA in water | 100 % MeOH | 0.3 | 80 | 25 | NA | 16 |
| Domingues et al., 2015 | MS | HILIC | 100 × 4.6 | 2.7 | 10mmol/L AA | ACN, 10 % 10mmol/L AA (60:40, v/v) | 0.5 | 5 | NR | NA | NR |
| Doolin et al., 2018 | MS | NR | NR | NR | NR | NR | NR | NR | NR | NA | NR |
| Eniu et al., 2019 | MS | AAA-MS | 250 × 3.0 | NR | AA in water | 10mM AF in MeOH | 0.25 | 5 | 35 | NA | 24 |
| Fukushima et al., 2014 | MS | C_18_ | 250 × 2.0 | 5 | Water/ACN (80:20), 0.1 % Acetic Acid | ACN/water (80:20), 0.1% Acetic Acid | 0.16 | 50 | 40 | NA | NR |
| Furtado et al., 2017 | MS | C_18_ | 75 × 2.1 | 1.7 | 0.2 % FA in water | 0.2 % FA in ACN | 0.9 | 5 | 50 | NA | 5 |
| Galla et al., 2021 | MS | C_18_ | 150 × 2.1 | 5 | 0.2 % FA in water | 02 % FA in ACN | 0.6 | 15 | 15 | NA | NR |
| Gomez-Gomez et al., 2017 | MS | C_18_ | 100 × 2.1 | 1.7 | NR | NR | 0.3 | NR | NR | NA | NR |
| Hajsl et al., 2020 | MS | C_18_ | 150 × 2.1 | 5 | 0.1 % FA | 0.1 % FA/ACN (v/v) | 0.25 | 20 | 40 | NA | 20 |
| Han et al., 2018 | MS | CR-I(+) | 150 × 3.0 | 5 | Ethanol, Water, TFA (50:50:0.4, v/v/v/) | ACN, TFA (100:0.4, v/v) | 0.4 | 5 | 15 | NA | 13 |
| Henykova et al., 2016 | MS | C_18_ | 100 × 2.1 | 1.8 | 0.1 % FA | MeOH | 0.3 | 10 | 30 | NA | NR |
| Huang et al., 2022 | MS | NR | NR | NR | NR | NR | NR | NR | NR | NA | NR |
| Huang et al., 2021 | MS | C_18_ | 150 × 4.6 | 3.5 | Water | ACN | 0.42 | 5 | 31 | NA | 24 |
| Jang et al., 2022 | MS | C_18_ | 100 × 2.1 | 1.8 | 0.1 % FA in water | ACN | 0.5 | 5 | 40 | NA | NR |
| Kim et al., 2015 | MS | C_18_ | 100 × 2.1 | 3 | 0.2 % Acetic Acid in water | 0.2 % Acetic Acid in ACN | 0.2 | 3 | 40 | NA | NR |
| Klatt et al., 2021 | MS | C_18_ | 50 × 2.1 | 1.8 | 0.1 % FA in water | 0.1 % FA in ACN | NR | NR | NR | NA | NR |
| Kurgan et al., 2022 | MS | C_18_ | 10 × 2.1 | 3 | Su (0.1 % FA) | MeOh Su (0.1 % FA) | 0.3 | 10 | 15 | NA | NR |
| Leichtle et al., 2012 | MS | NR | NR | NR | 1/1 Visopropanol/water | NR | 0.08 | 25 | NR | NA | NR |
| Lim et al., 2017 | MS | C_18_ | 100 × 2.1 | 1.8 | 50mM SA, 25mM ZA, 2.25 % ACN | 10 % ACN | 0.75 | 20 | 38 | NA | NR |
| Lionetto et al., 2021 | MS | F5 | 100 × 2.1 | 2.6 | 0.2 % FA | 100 % ACN | 0.3 | 10 | NR | NA | NR |
| Lu et al., 2019 | MS | C_18_ | 100 × 2.1 | 1.7 | 30 % ACN, 0.1 % FA, 10mmol/L AF | 95 % ACN, 0.1 % FA, 10mmol/L AF | 0.5 | 5 | 40 | NA | NR |
| Malhotra et al., 2017 | MS | NR | NR | NR | NR | NR | NR | NR | NR | NR | NR |
| Meng et al., 2022 | MS | HILIC | 150 × 2.1 | 2.6 | 75 % ACN, 7.5 % AF, 0.5 % FA | NR | 0.25 | 2 | 20 | NA | NR |
| Michel et al., 2020 | MS | C_18_ | NR | NR | Water | 0.1 % FA in ACN | NR | NR | NR | NA | 8 |
| Mierzchala et al., 2020 | MS | C_18_ | 50 × 1.0 | 1.8 | 0.1 % FA in water | 0.1 % FA in ACN | 0.07 | 3 | 50 | NA | NR |
| Nakatsukasa et al., 2011 | MS | C_8_ | 50 × 1.2 | 3 | 25mM FA | 60 % ACN in water | 0.25 | 5, 2 | 40 | NA | NR |
| Naz et al., 2019 | MS | C_18_ | 100 × 2.1 | 1.8 | 2.1 % FA in water | 0.1 % FA in 95 % ACN | 0.3 | 7.5 | NR | NA | NR |
| Oh et al., 2017 | MS | T3 | 100 × 2.1 | 3 | 0.1 % FA | 0.1 % FA in ACN | 0.2 | NR | 30 | NA | NR |
| Ohashi et al., 2013 | MS | C_18_ | 250 × 2.0 | 5 | Water, ACN (80:20), 0.1 % Acetic Acid | ACN, Water (80:20), 0.1 % Acetic Acid | 0.16 | 50 | 40 | NA | NR |
| Onesti et al., 2019 | MS | NR | NR | NR | NR | NR | NR | NR | NR | NR | NR |
| Panitz et al., 2021 | MS | C_18_ | 150 × 3.6 | 3 | 0.1 % FA in water | 0.1 % FA in ACN | 0.3 | 2 | NR | NR | NR |
| Primiano et al., 2020 | MS | NR | NR | NR | NR | NR | NR | NR | NR | NA | NR |
| Rodrigues et al., 2021 | MS | NR | TRP: 100 × 3.0  KYN: 150 × 2.1 | TRP: 2.5  KYN: 3 | 0.1 % FA in water | 0.1 % FA in ACN | TRP: 0.3  KYN: 0.2 | NR | TRP: 5  KYN: 25 | NA | NR |
| Ruoppolo et al, 2014 | MS | C_18_ | 150 × 4.6 | 5 | NR | NR | NR | NR | NR | NA | NR |
| Saito et al., 2022 | MS | PFP | 100 × 2.1 | 1.9 | 0.3 % FA in water | 0.3 % FA in MeOh | 0.3 | 5 | 45 | NA | 10 |
| Schwieler et al., 2020 | MS | C_18_ | 150 × 2.1 | 1.8 | 0.6 % FA in water | 0.6 % FA in MeOh | 0.3 | 3 | 50 | NA | 13 |
| Shi et al., 2019 | MS | C_18_ | NR | NR | Methanol, 0.1 % FA in water (95:5, v/v) | NR | 0.2 | 10 | 25 | NA | 4 |
| Smolenska et al., 2020 | MS | Hydro-RP | 50 × 2.0 | 2.5 | 0 % to 60 % ACN | NR | 0.2 | NR | 275 | NA | 12 |
| Sorgdrager et al., 2017 | MS | C_18_ | 100 × 2.1 | 3 | 0.2 % FA in water | ACN | 0.3 | NR | 25 | NA | NR |
| Sorgdrager et al., 2019 | MS | C_18_ | 100 × 2.1 | 2.5 | NR | NR | NR | 0.15 | NR | NA | NR |
| Sun et al., 2021 | MS | C_18_ | 50 × 4.6 | 2.7 | 80 % MeOh/20 % water, 2.5 mM AF in MeOh | NR | 1.6 | 15 | 20 | NA | NR |
| Sun et al., 2020 | MS | C_18_ | 100 × 2.1 | 1.7 | 10mmol/L Ammonium Acetate, 0.1 % FA in water | 0.1 % FA in ACN | 0.3 | 10 | 40 | NA | 10 |
| Suzuki et al., 2012 | MS | T3 | 150 × 2.1 | 5 | 5mM AF with 0.01 % TFA, MeOh (80:20, v/v) | 0.2 | NR | NR | NR | NA | NR |
| Suzuki et al., 2010 | MS | C_18_ | 150 × 0.5 | 3 | 2.1 % FA | 2.1 % FA, 40 % ACN | 0.012 | NR | NR | NA | NR |
| Suzuki et al., 2011 | MS | C_18_ | 150 × 0.5 | 3 | 2.1 % FA | 2.1 % FA, 40 % ACN | 0.012 | NR | NR | NA | NR |
| Taherizadeh et al., 2020 | MS | NR | NR | NR | NR | NR | 1 | NR | NR | NA | 60 |
| Tezcan et al., 2022 | MS | C_18_ | 50 × 4.6 | 5 | 0.1 % FA in water (v/v) | 0.1 % FA in ACN (v/v) | NR | 30 | 350 | NA | 5 |
| Tong et al., 2018 | MS | C_18_ | 50 × 2.1 | 5 | 0.1 % FA | 0.1 % FA in ACN | 0.5 | 5 | 40 | NA | 3.5 |
| Trepci et al., 2021 | MS | C_18_ | 150 × 2.1 | 1.8 | 0.6 % FA in water | 0.6 % FA in MeOh | 0.3 | NR | 50 | NA | 13 |
| Tuka et al., 2021 | MS | PFP | NR | NR | FA in water, MeOh | NR | NR | NR | NR | NA | NR |
| Van Faassen et al., 2019 | MS | C_18_ | 150 × 2.0 | 3 | 10mmol/L AA in 0.1 % FA | 0.1 % FA in 95 % ACN | 0.3 | 50 | NR | NA | 8.5 |
| Walser et al, 1993 | MS | C_18_ | 250 × 4.6 | NR | 0.02M Sodium Phosphate | MeOh | NR | NR | NR | NA | NR |
| Wang et al., 2019 | MS | C_18_ | 150 × 4.6 | 5 | 0.1 % FA in water, 0.1 % HFBA | 0.1 % FA in ACN, 0.1 % HFBA | 0.8 | 1 | 50 | NA | NR |
| Wang et al., 2018 | MS | PFP | 150 × 2.1 | 2.5 | 0.1 % FA in water | MeOh | 0.230 | 10 | 15 | NA | NR |
| Widner et al., 2000 | MS | NR | NR | NR | NR | NR | NR | NR | NR | NA | NR |
| Wu et al., 2022 | MS | C_18_ | 150 × 2.1 | 5 | NR | NR | NR | 10 | NR | NA | NR |
| Wu et al., 2020 | MS | NR | NR | NR | NR | NR | NR | NR | NR | NA | NR |
| Wu et al., 2018 | MS | NR | NR | NR | NR | NR | NR | NR | NR | NA | NR |
| Xu et al., 2012 | MS | C_18_ | 150 × 4.6 | 5 | 0.2 % FA, 0.005 % HFBA | 0.1 % FA in ACN, 0.005 % HFBA | 0.8 | NR | 50 | NA | NR |
| Yan et al., 2017 | MS | C_18_ | 100 × 2.1 | 2.7 | 0.2 % FA in water (v/v) | 0.1 % FA in ACN (v/v) | 0.18 | NR | NR | NA | 8.5 |
| Yilmaz et al., 2020 | MS | NR | NR | NR | NR | NR | NR | NR | NR | NA | NR |
| Zhang et al., 2020 | MS | C_18_ | 150 × 4.6 | 5 | 5mM AF in MeOh/water (45:55, v/v) | NR | 0.5 | NR | 35 | NA | NR |
| Zhou et al., 2022 | MS | C_18_ | 150 × 4.6 | 5 | MeOh/water (45:55, in 0.005mol/L AF) | MeOh/water (35:65, in 0.005mol/L AF) | 0.5 | NR | 35 | NA | NR |
| Zhou et al., 2019 | MS | C_18_ | 150 × 4.6 | 5 | MeOh/water (45:55, in 5mM AF) | MeOh/water (35:65, in 5mM AF) | 0.5 | NR | 35 | NA | NR |
| Barry et al., 2009* | PDA | C_18_ | 150 × 2.0 | 2 | 50mM Acetic Acid, 100mM ZA, 3 % (v/v ACN) | NR | 0.3 | NR | NR | FL: Ex/Em 254/404  PDA: 210 – 400 | NR |
| Al Saedi et al., 2022* | UV-Vis | C_18_ | 150 × 2.1 | 1.8 | 0.2mM SA | NR | 0.75 | 20 | 38 | KYN: 365  TRP: Ex/Em 280/438 | 12 |
| Bizzarri et al., 1990 | UV-Vis | C_18_ | 250 × 4.6 | 5 | ACN, SA buffer (pH 4.76; 4:96, v/v) | NR | 0.8 – 2.0 | 20 | NR | 280 | 24 |
| Buczko et al., 2007 | UV-Vis | C_18_ | 150 × 4.6 | NR | ACN, 0.1M AA, pH 4.65 | NR | 1.5 | NR | NR | 365 | NR |
| Capuron et al., 2011* | UV-Vis | C_18_ | 244 | 5 | 0.015mol/L Degassed PP (pH 6.4) | 27mL/L ACN | 0.8 | NR | 25 | KYN: 360  TRP: Ex/Em 285/365 | NR |
| Chen et al., 2010* | UV-Vis | C_18_ | NR | NR | 0.1M AA (pH 4.65) | NR | 1 | 30 | 22 | KYN: 360  TRP: Ex/Em 285/365 | NR |
| Clarke et al., 2009* | UV-VIS | C_18_ | 150 × 2.0 | 2 | 50mM Acetic Acid, 100mM ZA, 3 % (v/v) ACN | NR | 0.3 | 20 | 30 | KYN: 330  TRP: Ex/EM 254/404 | 30 |
| Crotti et al., 2019* | UV-VIS | C_18_ | 250 × 4.6 | 5 | ACN, 0.005 Phosphate Buffer (15:85 v/v) | NR | 1 | NR | NR | KYN: 360  TRP: Ex/Em 285/345 | NR |
| Frick et al., 2004* | UV-Vis | C_18_ | NR | NR | NR | NR | 0.9 | NR | NR | KYN: 360  TRP: Ex/Em 285/365 | NR |
| Geisler et al., 2015* | UV-Vis | C_18_ | NR | 5 | 15mmol/L Acetic Acid-SA (pH 4.0) | NR | NR | NR | NR | KYN: 360  TRP: Ex/Em 286/366 | NR |
| Girgin et al., 2020* | UV-Vis | NR | NR | NR | 15mM pH 6.5 Monopotassium Phosphate, 0.7 % ACN | NR | 0.8 | NR | NR | KYN: 360  TRP: Ex/Em 286/Em | NR |
| Gulaj et al., 2010* | UV-Vis | C_18_ | 150 × 2.1 | NR | KYN: 0.1M Acetic Acid, 0.1M AA, 2 % ACN  TRP: 50mM Acetic Acid, 0.25M ZA, 1.2 % ACN | NR | 0.2 | NR | NR | KYN: 365  TRP: Ex/Em 254/404 | NR |
| Islam et al., 2020 | UV-Vis | C_18_ | NR | NR | 0.1 % FA in water | 0.1 % FA in ACN | 0.8 | 5 | NR | NR | NR |
| Kim et al., 2009* | UV-Vis | C_18_ | 100 × 4.7 | NR | TRP: 57.2g Na2HPO412H2O, 60mL ACN in water  KYN: 250mM ZA in water | TRP: 420 water/280 CAN/320 MeOh  KYN: 9 % ACN | NR | NR | NR | KYN: 365  TRP: Ex/Em 340/440 | NR |
| Lorite et al., 2007* | UV-Vis | C_18_ | 244 × 4.0 | 5 | Phosphate | NR | 0.8 | 100 | NR | KYN: 360  TRP: Ex/Em 285/365 | NR |
| Ma et al., 2009 | UV-Vis | C_18_ | 125 × 4.0 | 5 | 50mmol/L SA, 6 % ACN (v/v) | NR | 0.8 | 20 | 25 | KYN: 360  TRP: 302 | NR |
| Myint et al., 2007* | UV-Vis | C_18_ | 100 × 4.7 | 5 | 50mM ZA, Acetic Acid, 1.0 % ACN | NR | 1 | 100 | NR | KYN: 365  TRP: Ex/Em 340/440 | NR |
| Myint et al., 2007* | UV-Vis | C_18_ | 100 × 4.7 | NR | TRP: 57.2g NA2HPO412H2O, 160mL ACN in water  KYN: 250mM ZA in water | TRP: 420 water/280 ACN/320 MeOh | NR | NR | NR | KYN: 365  TRP: Ex/Em 340/440 | NR |
| Palabiyik et al., 2016* | UV-Vis | C_18_ | 250 × 4.6 | 5 | 15mM Phosphate, 27mL/L ACN | NR | 0.8 | 100 | NR | KYN: 360  TRP: Ex/Em 286/366 | NR |
| Pertovaara et al., 2005* | UV-Vis | C_18_ | 50 × 2.1 | 5 | 15mmol/L SA in Acetic Acid, 27mL/L ACN | NR | 0.9 | 10 | NR | KYN: 360  TRP: Ex/Em 266/366 | NR |
| Roca et al., 1999 | UV-Vis | C_18_ | 150 × 3.9 | 4 | 0.14M SA, 0.5mL/L TEA | 60 % ACN in water | NR | NR | NR | 254 | NR |
| Saito et al., 1979* | UV-Vis | CK-10-S | 300 × 4.6 | NR | NR | NR | 0.73 | NR | 60 | KYN: 280  TRP: Ex/Em 280/340 | 60 |
| Souissi et al., 2022 | UV-Vis | C_18_ | 150 × 4.6 | 5 | 15mM Phosphate Buffer, 10.6 % ACN | NR | 1.2 | NR | 30 | KYN: 360  TRP: 280 | KYN: 3  TRP: 5 |
| Sousa et al., 2021* | UV-Vis | C_18_ | NR | 3 | 20mM AF in 0.01 % FA in water, ACN, Ethanol (95/2/3, v/v/v) | NR | 0.7 | 10 | 25 | KYN: 365  TRP: Ex/Em 280/348 | 27 |
| Sultana et al., 2012 | UV-Vis | C_18_ | 250 × 4.6 | NR | Water, ACN (90:10, v/v) | NR | 1.5 | 20 | 25 | 273 | NR |
| Zhao et al., 2011* | UV-Vis | C_18_ | 250 × 4.6 | 5 | 20mmol/L NaAc, 30mmol/L HAc, 3 % MeOh | 20mmol/L NaAc/HAc, 10 % MeOh, 19 % ACN | 1 | 50 | 25 | KYN: 365  TRP: Ex/Em 292/340 | 30 |
| Zhen et al., 2011 | UV-Vis | C_8_ | 150 × 4.6 | 5 | 10mmol/L Acetate Buffer, ACN (94:6, v/v) | NR | 0.6 | 25 | 25 | KYN: 360  TRP: 302 | 8 |
| *Note:* AA: Ammonium Acetate; ACN: Acetonitrile; AF: Ammonium Formate; Ex/Em: Excitation/Emission; FA: Formic Acid; HFBA: Heptafluorobutyric Acid; MeOh: Methanol; PFP: Pentafluorophenyl; PP: Potassium Phosphate; SA: Sodium Acetate; TFA: Trifluoroacetic Acid; ZA: Zinc Acetate. | | | | | | | | | | | |
